# *Staphylococcus aureus* and Methicillin Resistant *S. aureus* in Nepalese Primates: Resistance to Antimicrobials, Virulence, and Genetic Lineages

**DOI:** 10.3390/antibiotics9100689

**Published:** 2020-10-13

**Authors:** Marilyn C. Roberts, Prabhu Raj Joshi, Stefan Monecke, Ralf Ehricht, Elke Müller, Darius Gawlik, Celia Diezel, Sascha D. Braun, Saroj Paudel, Mahesh Acharya, Laxman Khanal, Narayan P. Koju, Mukesh Chalise, Randall C. Kyes

**Affiliations:** 1 Department of Environmental and Occupational Health, School of Public Health, University of Washington, Seattle, WA 98105, USA; 2 Nepalese Farming Institute, Maitidevi, Kathmandu 44600, Nepal; cmilanjoshi@gmail.com (P.R.J.); pulu.saroj@gmail.com (S.P.); maheshacharya045@gmail.com (M.A.); 3 Leibniz Institute for Photonic Technologies Leibniz-IPHT), 07745 Jena, Germany; stefan.monecke@leibniz-ipht.de (S.M.); ralf.ehricht@leibniz-ipht.de (R.E.); elke.mueller@leibniz-ipht.de (E.M.); Celia.diezel@leibniz-ipht.de (C.D.); sascha.braun@leibniz-ipht.de (S.D.B.); 4 InfectoGnostics Research Campus Jena, 07743 Jena, Germany; 5 Institute for Medical Microbiology and Hygiene, Medical Faculty “Carl Gustav Carus”, Technische Universität Dresden, 01062 Dresden, Germany; 6 Institute of Physical Chemistry, Friedrich-Schiller University, 07743 Jena, Germany; 7 PTC—Phage Technology Center GmbH, 59199 Bönen, Germany; darius.gawlik@web.de; 8 Central Department of Zoology, Institute of Science and Technology, Tribhuvan University, Kathmandu 44613, Nepal; lkhanal@cdztu.edu.np; 9 Center for Postgraduate Studies, Nepal Engineering College, Pokhara University, Lalitpur 44800, Nepal; npkoju.2003@gmail.com; 10 Department of Psychology, University of Washington, Seattle, WA 98195, USA; 11 Nepal Biodiversity Research Society and Central Department of Zoology, Tribhuvan University, Kirtipur, Kathmandu 44613, Nepal; mukesh57@hotmail.com; 12 Washington National Primate Research Center, Center for Global Field Study, Departments of Psychology, Global Health, Anthropology, University of Washington, Seattle, WA 98195, USA; rkyes@uw.edu

**Keywords:** *Staphylococcus aureus*, methicillin resistant *S. aureus*, antibiotic resistance genes, virulence factors, genetic lineages

## Abstract

*Staphylococcus aureus* is a ubiquitous pathogen and colonizer in humans and animals. There are few studies on the molecular epidemiology of *S. aureus* in wild monkeys and apes. *S. aureus* carriage in rhesus macaques (*Macaca mulatta*) and Assam macaques (*Macaca assamensis*) is a species that has not previously been sampled and lives in remote environments with limited human contact. Forty *Staphylococcus aureus* isolates including 33 methicillin-susceptible *S. aureus* (MSSA) and seven methicillin-resistant *S. aureus* (MRSA) were characterized. Thirty-four isolates were from rhesus macaques and six isolates (five MSSA, one MRSA) were from Assam macaques. Isolates were characterized using StaphyType DNA microarrays. Five of the MRSA including one from Assam macaque were CC22 MRSA-IV (PVL+/*tst*+), which is a strain previously identified in Nepalese rhesus. One MRSA each were CC6 MRSA-IV and CC772 MRSA-V (PVL+). One MSSA each belonged to CC15, CC96, and CC2990. Six MRSA isolates carried the *blaZ*, while ten known CC isolates (seven MRSA, three MSSA) carried a variety of genes including *aacA-aphD*, *aphA3, erm*(C), *mph*(C), *dfrA*, *msrA*, and/or *sat* genes. The other 30 MSSA isolates belonged to 17 novel clonal complexes, carried no antibiotic resistance genes, lacked Panton–Valentine Leukocidin (PVL), and most examined exotoxin genes. Four clonal complexes carried *egc* enterotoxin genes, and four harbored *edinB*, which is an exfoliative toxin homologue.

## 1. Introduction

*Staphylococcus aureus* is a ubiquitous pathogen and colonizer in humans and in a variety of wild and domestic animals [1,2]. However, there are only a few studies on its presence, and molecular epidemiology, in monkeys and apes. In earlier studies, we characterized methicillin-resistant *S. aureus* (MRSA) isolates from nasal samples in a United States Primate Center [2]. Three species of macaques [*Macaca mulatta, M. fascicularis*, and *M. nemestrina*] were identified with an MRSA as well as a few methicillin-susceptible *S. aureus* (MSSA) isolates [2,3]. Two different MRSA clones were identified as having a previously uncharacterized sequence type (ST3268) and ST188, which is a rare ST in the United States but more common among humans from Southeast Asia [2,3] and wild animals [1]. Both clones have been identified in other United States primate facilities as well as commercial facilities [1,3 and unpublished observations]. The isolates were characterized by PCR, multilocus sequence typing (MLST), whole genome sequencing, single -nucleotide polymorphism [SNP] analysis, pulsed-field gel electrophoresis (PFGE) and microarray-based assays [2,3,4]. The data suggested an importation of the MRSA strains together with the primates from outside rather than an introduction by local staff members [2,3]. 

**Figure 1:**
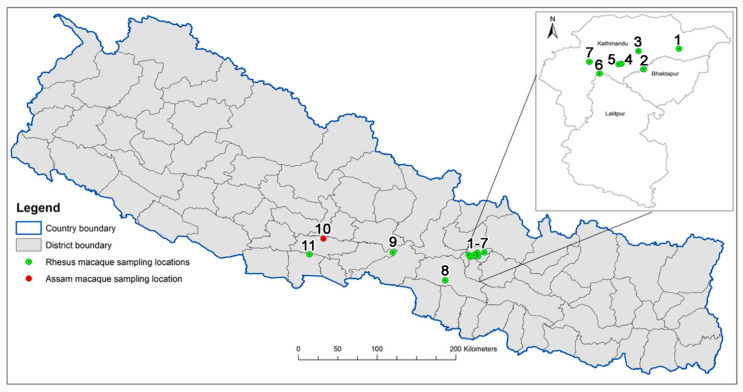
Primate Sampling Locations. Saliva Sampling was conducted at 11 locations in Nepal: 1-Bajrayogini; 2-Nilbarahi; 3-Gokarna; 4-Guheswari; 5-Pashupati; 6-Thapathali; 7-Swayambhu; 8-Hetauda; 9-Chitwan; 10-Ramdi; 11-Rupandehi.

Therefore, it was of interest to look at wild *Macaca* spp. to determine if either ST188 or ST3268 were carried by these primates as well. We did two studies in Nepal characterizing MRSA isolates cultured from wild rhesus macaques (*Macaca mulatta*) saliva samples [4,5]. The animals were living in and around temple areas of the Kathmandu valley in Nepal, where human–macaque interaction is common. In this first study, we found four MRSA belonging to two sequence types (ST), 22 SCC*mec* type IV and CC239 SCC*mec* type III. Both lineages are known pandemic MRSA lineages. ST239-MRSA-III is a hospital-associated strain that spread globally for the last five decades. CC22-MRSA-IV comprises several related strains with different SCC*mec* IV subtypes and toxin genes profiles [4,5,6]. This includes EMRSA-15, which is a common and widespread strain in hospital and community settings especially in Western Europe. It also includes a strain that harbors PVL and *tst1* genes emerging in Arabian Gulf countries that appear to be identical by microarray with regard to a rare combination of Panton–Valentine Leukocidin (PVL) and the toxic shock (*tst-1*) gene and by SCC*mec* IV subtype to the Nepalese isolates. CC22-MRSA were first identified in Nepal from hospitalized patients in 2012 [7], although other studies did not identify CC22 in hospital samples [8]. The data from the previous work led us to hypothesize that humans were a likely source of the CC22-MRSA and CC239-MRSA isolates in the wild Nepalese macaques. In the second study, we collected saliva samples from *Macaca mulatta* as well as environmental samples from five surrounding areas, including the Bajrayogini temple site outside the Kathmandu valley [4] (Figure 1). Thirteen MRSA were isolated from primates, 19 were isolated from the environment, and 5 were taken, for comparison, from humans. Four (31%) of the primate isolates, 14 (74%) of the environmental, and the five human MRSA isolates were all CC22 SCC*mec* IV, as found in the first study [5]. Sixteen (89%) carried both the PVL and the *tst-1* gene, which is an unusual combination that according to the microarray profiles is the same as in the previously characterized strains from both Nepalese macaques and pigs [5]. Most of the strains from both the primates and environment are known from earlier studies to be associated with humans [4,5].

In the current study, we wanted to focus on MSSA isolates from these primates in order to elucidate if their native MSSA population also was directly related to those in humans. This has been previously the case of MSSA isolated from great apes and lemurs in a wildlife sanctuary in Africa, where human interaction was significant compared to that found typically in the US Primate Centers and zoos [1,9]. We also wanted to look at a primate population with limited human interaction and a different host species than has previously been sampled and thus, we chose to sample Assam macaques (*Macaca assamensis*). This would allow us to determine if MSSA and MRSA isolates are most likely acquired from humans in the Assam macaque’s population or if there is a potential to have primate-specific MSSA and/or MRSA. We included a few MRSA from areas previously sampled to verify that the clones had not changed over the years, since our first and second set of isolates were taken as well as new areas not previously sampled. 

## 2. Results

### 2.1. CC for MSSA Typing and CC and SCCmec Typing for MRSA Ancestral Lineage

The sampling locations, the primate host species, and the SCC*mec* typing data are provided for the seven MRSA, which includes one MRSA from an Assam macaque sample and six isolates from rhesus macaque samples. The same information without the SCC*mec* typing data are provided for the 33 MSSA isolates, including five MSSA sampled from the Assam macaques and 28 sampled from the rhesus macaques (Table 1).

There were ten (25%) isolates that could be assigned to known lineages (Table 1). This includes all seven MRSA isolates including one isolated from the Assam macaque and three MSSA isolates [2 rhesus and 1 Assam] (Table 1). The other 30 MSSA isolates were not able to be clonally characterized using the StaphyType DNA microarrays and the underlaying database, although experiments were valid. This indicated affiliation to previously unknown lineages (Supplementary Table S1 MLST Sequences). The presence of genes of the Staphyloxanthin operon (*crtM, crtN, crtO, crtP*, as established by a second array-based assay for one isolate of each strain) ruled out an identification as *Staphylococcus argenteus*. Strong positive reactivity with probes for *coA*, *nuc1*, and *sbi* ruled out *Staphylococcus schweitzeri*.

### 2.2. Antibiotic Resistance Genes

Antibiotic resistance gene carriage, based on the microarray, is shown in Table 2 for all 40 isolates. The antibiotic susceptibility patterns are shown in Table 3 with the actual MIC data in Supplementary Table S2. The microarray detection of specific antibiotic resistance genes and the phenotypic susceptibility patterns are correlated with the MSSA isolates that could not be assigned to known lineage while also being susceptible to all 20 antibiotics tested. Thirty of 33 (91%) MSSA isolates, which includes all of the isolates for which we could not determine their clonal lineage, did not carry any of the 33 detected antibiotic resistance genes often associated with MSSA and MRSA isolates (*aacA-aphD*, *aadD*, *aphA3*, *blaZ*, *cat*, *cfr*, *dfrA*, *erm*(A), *erm*(B), *erm*(C), *far1*, *fexA*, *fusC*, *lnu*(A), *mecA*, *mecC*, *mef*(A), *msr*(A), *mph*(C), *mupR*, *sat*, *tet*(K), *tet*(M), *qacA*, *qacC*, *vga*(A), *vanA*, *vanB*, *vanZ*, *vga*(B), *vgb*(A), *vat*(A), and *vat*(B)), which were detected by the StaphyType DNA microarrays. The remaining three MSSA and all seven MRSA carried a variety of antibiotic resistance genes. All MRSA isolates carried one to six different antibiotic resistance genes besides the *mecA* gene. The CC722-MRSA-V carried six of the eight antibiotic resistance genes identified in the MRSA and MSSA isolates. 

CC06 MRSA-IV isolate carried, besides *mecA*, only the *erm*(C) macrolide–lincosamide–streptogramin B resistance gene (Table 2). The CC22-MRSA-IV isolates carried *blaZ* (beta-lactamase) and the bifunctional aminoglycoside resistance gene *aacA-aphD.* The *dfrA* gene coding for co-trimoxazole resistance was present in all five isolates, but it was not always expressed; two isolates were susceptible. The *erm*(C) gene was found in four out of five isolates; it translated into inducible clindamycin resistance. The MRSA isolate #97 from the Assam macaque had the same antibiotic resistance profile as three of the four other CC22 MRSA-IV [isolates #93, #94, #C] isolated from rhesus macaque samples in areas with more human contact. (Table 2 and Table 3).

The CC772-MRSA carried *aacA-aphD*, the *mph*(C) macrolide resistance gene, and the *msr*(A) macrolide and streptogramin B resistance gene. It also harbored both *aph3*, coding aminoglycoside resistance, and *sat*, coding for streptothricin resistance, which is a combination that is frequently present in CC772 “Bengal Bay Clone” isolates [10]. 

Three MSSA isolates [CC15 Rhesus, CC96 Assam, CC2990 Rhesus] carried between one and two different antibiotic resistance genes. The CC15 [#83] carried the *blaZ* and the *erm*(C) genes, while CC96 [#89] carried the *msr*(A) and the CC2990 isolate [H] was positive for the *aacA-aphD* gene. The remaining 30 MSSA regardless of where they were collected and whether they were from Assam or rhesus macaques carried none of the antibiotic resistance genes covered by the microarray assays, and apart from intermediate resistance to nitrofurantoin, they did not show phenotypic resistance to the compounds tested (Table 3). 

### 2.3. Accessory and Virulence Factors

The presence of important virulence factors is summarized in Table 4. The five CC22 MRSA-IV carried most notably the PVL and *tst-1* genes (Table 1 and Table 4). Thus, it is the same clone as identified in previous work on MRSA carriage in Nepalese rhesus macaques [4,5]. The CC772-MRSA-V harbored not only PVL but also the *egc* enterotoxin cluster genes, enterotoxins *sea*, *sec*, and *sel* as well as an enterotoxin homologue ORF CM14. This is in accordance with previous descriptions of that strain [10,11,12]. The CC96 MSSA from the Assam macaque had virulence-associated genes *lukD/E*, *cna*, and *sasG*. The CC15 MSSA carried virulence-associated genes *lukD/E*, *chp*, and *scn*, which is in accordance with previously described human isolates [13]. 

CC2990 is a poorly known clonal complex. One isolate, from a rhesus, was found to harbor *sec+sel*, *lukD/E*, *sak*, *chp*, *scn*, *edinB+etD2*, *cna*, and *sasG*. The other MSSA isolates from both primate species had different sets of virulence-associated genes (Table 4); two of these 17 lineages carried the *egc* cluster; four harbored *edinB* (epidermal cell differentiation inhibitor B/ADP-ribosyltransferase; GenBank AB057421.1, 7154 to 7897) and an exfoliative toxin homologue *etD2* (GenBank HF563069), which is a rare combination among previously studied strains (see Discussion). 

## 3. Discussion

The *S. aureus* isolates from free-ranging Nepalese macaques could roughly be divided into three categories. First, there were MRSA strains that frequently have been observed also in humans, in Nepal, the Indian subcontinent, and in the Arabian Gulf states [6,7]. The CC6-MRSA-IV and CC22-MRSA-IV (PVL+/*tst*+) are human MRSA with strong Middle Eastern connections [6]. CC772-MRSA-V (PVL+), “Bengal Bay Clone”, is an epidemic strain emerging from the Indian subcontinent, being common in India, Pakistan, and Bangladesh, as well as in the Gulf States where there are many Indian/Pakistani and Nepalese working migrants [10,11,12,13]. It might be speculated that Nepalese expatriates brought these strains from the Arabian Gulf states into Nepal, triggering local outbreaks among humans and, via food and sacrificial offering at temples, among macaque populations [14]. 

Second, there are MSSA strains that might come from humans or from animals/livestock, although data for some of these lineages are not sufficient to assess their host specificity and geographic distribution. The MSSA CC15 is a common lineage among humans [15]. MSSA CC96 is a rare lineage among humans, with a few reports on MRSA from Malaysia, Central Asia, and Middle East [16]. It has also previously been isolated from rabbits [17], so that it cannot be decided if it was primarily a zoonotic or anthropozoonotic lineage. One isolate was assigned to CC2990 based on both the microarray profile and MLST, matching some Western European isolates of this lineage; however, these were PVL-positive (unpublished observation by the authors). 

Third, the remaining 17 lineages comprising 30 MSSA isolates did not match with any known array profile, neither human nor known animal ones; thus, no CC could be assigned (Supplementary Table S1 MLST Sequences) and no information on origin, host specificity, and geographic distribution is available. In contrast to the other lineages, from this as well as from the previous studies [4,5], they did not carry commonly found antibiotic resistance genes and did not display phenotypic resistance. With regard to virulence factors, there was a conspicuous presence of *edinB* and *etD2* in four of the unknown lineages as well as in CC2990. The *edinB* gene is rare among human isolates, and it is usually linked to an exfoliative toxin homologue, *etD* (GenBank AB057421.1, 5409 to 6254). For instance, they can be found in the European community-acquired CC80-MRSA-IV clone. The divergent *etD2* exfoliative toxin homologue was previously identified in European sheep and hedgehogs (GenBank HF563069) [18,19]. The role of these virulence factors in macaques is not yet elucidated. These 17 lineages can preliminarily be considered as colonizers native to wild macaque populations. However, since virtually nothing is known on MSSA population structures among humans and livestock in remote Nepalese regions, this cannot currently be assessed. Further investigations on geographic distribution, host specificity, and possible relevance for animal/livestock and human health are warranted. 

We found the same strains circulating among Assam macaques and rhesus macaques (Table 1, Table 2, Table 3 and Table 4). Sample numbers are too low to detect possible differences in carriage between the two macaque species. However, this suggests that there could be a transmission between macaque species, and it strongly indicates a transmission of “human”, anthropozoonotic strains (such as CC22 and CC772-MRSA) from contaminated environments and occasional human or livestock contact to the Assam and rhesus macaques. How the colonization of MRSA impacts the carriage of MSSA in the same host should also be investigated, as well as the impact on the health of the primate hosts. It is not clear whether exposure to such anthropozoonotic MRSA strains might pose a danger to wild monkey populations, especially given the fact that Assam macaques are much more rare than rhesus. Luckily, PVL seems to be less effective as a cytotoxin in macaques (long-tailed macaque, *Macaca fascicularis*) than in humans [20]. However, there are no data on the ability to cause disease in wild monkeys, neither for these “human” and/or PVL-positive strains nor for the 17 novel macaque lineages. Conversely, it is not known if macaque-specific lineages can be passed to humans and if “human” strains, in Nepal and elsewhere, evolved from macaque strains. This can be elucidated only by genome-wide analysis of less known, local, human strains and those macaque strains. Furthermore, it should be determined if the MSSA lineages identified in the current study are primarily found in primates from Nepal or also in wild primates elsewhere and/or in other wild animals. It would also be of interest to determine if these MSSA isolates are carried with or without causing disease in either primates or humans. 

## 4. Materials and Methods

### 4.1. Primate Sampling

A total of 11 locations were sampled in the current study (Figure 1). The locations, all of which represent religious/temples sites, included Bajrayogini, Nilbarahi, Pashupati, Swayambhu [21], and Thapathali (these five locations were sampled in 2018 [5] and 2019 [4]). Additionally, six new locations were sampled including, Chitwan, Guheswari, Gokarna, Hetauda, Rupandehi, and Ramdi. All of the locations involved the sampling of rhesus macaques with the exception of Ramdi, where we sampled a resident group of Assam macaques consisting of about 32 individuals living around the Ramdi temple (West-Ramdi) [22]. Human dwellings are situated a few hundred meters from the temple. The diet of these macaques consists of food from the forest, but they also are fed fruits and other household scraps by the local people and pilgrims visiting the temple. These macaques are habituated to humans and have been reported to visit and raid human settlements and crop fields in the area [23]. The sampled macaques appeared healthy based on physical appearance and behavior.

The collection technique involved an adaptation of the non-invasive oral sampling method previously described [4,5,14] using SalivaBio Children’s Swabs (Salimetrics LLC, State College, PA, USA) [4,24]. Swabs were soaked in a sterile glucose solution (10% *w*/*v*) and thrown to the macaques. A new pair of disposable gloves was used before taking the swab out of the tube and providing it to the monkey. After chewing for several seconds/minutes, the monkey realized the swab was not food and discarded it. The storage tube contained enrichment broth Bacto^®^ m Staphylococcus Broth (Difco Laboratories, Sparks, MD, USA) supplemented with a final concentration of 75 mg/L of polymyxin B, 0.01% potassium tellurite, and either with or without 12.5 mg/L nystatin to prevent fungal growth (Sigma-Aldrich, St Louis, MO, USA). The tubes were returned to the laboratory the same day when possible and incubated at 37 °C until turbid (24–96 h), as previously described [25]. The broth was streaked for isolation onto mannitol salt agar plates (HiMedia Laboratories, Mumbai, India), and yellow colonies were sub-cultured onto blood agar plates (HiMedia Laboratories, Mumbai, India). Colonies that had β−hemolysis were verified as *S. aureus* as described below. Forty isolates, including 7 MRSA and 33 MSSA, were randomly selected from a total of 97 isolates (85 MSSA and 13 MRSA). One MRSA was selected from each region that had an MRSA isolate. All five of the MSSA from the Assam were included, while from the other regions, 28 randomly selected MSSA were included in the current study. 

### 4.2. Ethical Statement 

The research protocol for the sampling of free-ranging primates in Nepal was approved by the Department of Forest and Soil Conservation under the Ministry of Forest and Environment, Government of Nepal (Reference Letter Number: 075/076/663). This research also complied with the animal use protocol for primates (#3143-04) approved by the Institutional Animal Care and Use Committee at the University of Washington, USA, and the American Society of Primatologists (ASP) Principles for the Ethical Treatment of Nonhuman Primates. 

### 4.3. Identification of S. aureus from Primates 

Colonies that had β-hemolysis on blood agar plates were verified as *S. aureus* by Gram stain and with the Staphaurex test as previously described (Thermo Fisher Scientific Remel Products, Lenexa, KS [25]. MRSA isolates were identified by their ability to grow on Mueller–Hinton agar (HiMedia Laboratories, India) supplemented with 4 mg/L of oxacillin (HiMedia Laboratories, India). The MRSA isolates were confirmed using the Thermo Scientific PBP2′ latex agglutination test kit according to the manufacturer’s instructions (Thermo Fisher Scientific Remel Products, Lenexa, KS, USA) [25]. 

### 4.4. Antibiotic Susceptibility Testing 

The antimicrobial testing was done for 20 antibiotics: benzylpenicillin, oxacillin, cefoxitin, gentamicin, tobramycin, ciprofloxacin, levofloxacin, moxifloxacin, erythromycin, clindamycin, linezolid, teicoplanin, vancomycin, tetracycline, fosfomycin, nitrofurantoin, fusidic acid, mupirocin, rifampicin, and trimethoprim/sulfamethoxazole using automated microdilution, VITEK 2 by Biomerieux according to VITEK 2 by Biomerieux using the manufacturer’s instructions and utilizing European Committee on Antimicrobial Susceptibility Testing EUCAST breakpoints Table 2 [26]. 

### 4.5. DNA Microarray Analysis

The StaphyType (Abbott/Alere Technologies, Jena, Germany) DNA microarray-based assay was used to screen for a presence or absence of a multitude of genes, including antibiotic resistance markers, virulence factors, species-specific controls, and typing markers (Table 1, Table 3 and Table 4). This system has previously been used for a variety of studies on MRSA [3,4,11,19,20,27,28,29]. The microarray typing includes 334 target sequences and ≈170 separate genes and allelic variants including species markers, SCC*mec*, capsule, and *agr* group typing markers, which commonly carried staphylococcal antibiotic resistance genes, toxins, and microbial surface components recognizing adhesive matrix molecules [MSCRAMM] genes. Isolates were assigned to clonal complexes (CCs) by an automated comparison of the microarray hybridization profiles to a large database of previously characterized isolates [11]. Then, strains were assigned to clonal complexes when possible. However, 30 of the isolates could not be assigned to a clonal complex and were assumed to be novel (Supplementary Table S1). The detailed protocol as well as the sequences of primers and probes have previously been published [15]. A second array was used on representative isolates for the detection of Staphyloxanthin genes [30]. 

### 4.6. Multilocus Sequence Analysis

Genomic DNA was isolated from an overnight culture grown at 37 °C on Columbia Blood Agar plates (Becton Dickinson GmbH, Heidelberg, Germany) using a Macherey and Nagel NucleoSpin^®^ Microbial DNA kit (MACHEREY-NAGEL GmbH & Co. KG, Dueren, Germany). The Oxford Nanopore MinION platform was used for sequencing the whole genome of twelve isolates. Briefly, size selection and DNA clean-up were performed using Agencourt AMPure XP beads (Beckman Coulter GmbH, Krefeld, Germany) in a ratio of 1/1 (*v*/*v*). The DNA library was generated using the Nanopore native barcoding genomic DNA kit SQK-LSK109 in combination with the native barcoding expansion kit EXP-NBD104 (Oxford Nanopore Technologies, Oxford, UK) according to the manufacturer’s instructions. The used flow cell FLO-MIN106 (revD R9.4.1) was primed by the flow cell priming kit EXP-FLP001 (Oxford Nanopore, Oxford, UK). The protocol named “Native barcoding genomic DNA” was used in version NBE_9065_v109_revV_14Aug2019 (Last update: 21/02/2020). 

The Guppy basecaller (version 4.0.14+8d3226e, Oxford Nanopore Technologies, Oxford, UK) translated the MinION raw reads (FAST5) into quality tagged sequence reads (4000 reads per FASTQ-file) using the barcode trimming option. Flye (v2.8-b1674) was used to assemble the quality tagged sequence reads of each strain to one big circular contig. The polishing of assemblies was split into two steps. At first, racon (v1.4.17) was iteratively used four times with the following parameter: match 8; mismatch 6; gap 8; and windows-lengths 500. Afterwards, medaka (v1.0.3) (https://nanoporetech.github.io/medaka/) ran on the last racon polished assembly using the model r941_min_high_g360. Corrected assemblies were used for further MLST analysis by upload to the *S. aureus* pubMLST database (https://pubmlst.org/bigsdb?db=pubmlst_saureus_seqdef&page=sequenceQuery).

## 5. Conclusions

The MRSA isolated from both Assam and rhesus macaques were linked to other countries including India and Middle East as the previous study illustrated for MRSA isolated in 2017–2018 [4,5]. The same cannot be said for the majority of the MSSA. Thirty isolates representing 17 distinct lineages did not belong to previously characterized clonal complexes nor did they carry antibiotic resistance genes commonly found in *S. aureus*. This suggests that these isolates may not commonly circulate in humans and may be adapted for macaques. We did not find comparable MRSA to these novel MSSA, suggesting that the two may use different transmission routes in the primates. Future studies will need to expand the knowledge of MSSA in primates and determine if these isolates can be identified in domestic or other types of wild animals. The number of isolates from Assam macaques that have limited contact with humans suggest that they carry the same type of MRSA and MSSA as the rhesus. Therefore, it will be of interest to determine if both MRSA and MSSA are transmitted from Assam adults to their infants and how contaminated their environment is with MSSA.

## Figures and Tables

**Table 1:** Methicillin-resistant *S. aureus* (MRSA) strains, sample types, and sampling locations as well as species markers, *agr* groups, and capsule types.

Clonal Complex	Strain	Isolate ID	Number	Sampling Location	Host Species	*gapA*, *katA*, *coA*, *nuc1*, *spa*, *sbi*, *eno*, *clfA+B*, *fnbA*	Staphyloxanthin Operon	*agr* Group	Capsule Type
CC06	CC6-MRSA-IV	B	1	Nilbarahi	Rhesus	**POS**	not tested	**I**	**8**
CC22	CC22-MRSA-IV (PVL+/tst+)	93, 94, 05, 97, C	5	2 Gokarna, 1 each Chitwan, Ramdi and Nilbarahi	1 Assam, 4 Rhesus	**POS**	not tested	**I**	**5**
CC772	CC772-MRSA-V (PVL+), “Bengal Bay”	91	1	Hetauda	Rhesus	**POS**	not tested	**II**	**5**
CC15	CC15-MSSA	83	1	Rupandehi	Rhesus	**POS**	not tested	**II**	**8**
CC96	CC96-MSSA	89	1	Ramdi	Assam	**POS**	not tested	**III**	**8**
CC2990	CC2990-MSSA	H	1	Guheswari	Rhesus	**POS**	**POS**	**II**	**8**
UNKNOWN 01	ST(4-13-1-105-11-5-x)-MSSA	86, 87	2	Ramdi	Assam	**POS**	**POS**	**I**	**5**
UNKNOWN 02	ST(1-421-1-1-12-238-11)-MSSA	52, 53	2	Gokarna	Rhesus	**POS**	**POS**	**I**	**5**
UNKNOWN 03	ST(1-3-1-15-28-x-1)-MSSA	31, 32, 71, D	4	2 Nilbarahi, 1 each Chitwan and Gokarna	Rhesus	**POS**	**POS**	**I**	**8**
UNKNOWN 04	MSSA	01	1	Pashupati	Rhesus	**POS**	**POS**	**I**	**8**
UNKNOWN 05	ST(1-1-1-1-28-4-11)-MSSA	73, F	2	1 Chitwan, 1 Gokarna	Rhesus	**POS**	**POS**	**I**	**8**
UNKNOWN 06	ST(1-421-1-x-x-1-11)-MSSA	54	1	Gorkarna	Rhesus	**POS**	**POS**	**I**	**8**
UNKNOWN 07	ST(12-x-1-66-11-x-x)-MSSA	29, 30, 51	3	2 Swayambhu, 1 Gokarna	Rhesus	**POS**	**POS**	**II**	**8**
UNKNOWN 08	ST(1-38-1-1-x-238-x)-MSSA	81, 88	2	1 Ramdi, 1 Rupandehi	1 Assam, 1 Rhesus	**POS**	**POS**	**II**	**8**
UNKNOWN 09	ST(3-3-2-66-28-x-x)-MSSA	10	1	Pashupati	Rhesus	**POS**	**POS**	**II**	**8**
UNKNOWN 10	ST(3-1-1-66-28-1-x)-MSSA	82, 90	2	1 Ramdi, 1 Rupandehi	1 Assam, 1 Rhesus	**POS**	**POS**	**III**	**5**
UNKNOWN 11	MSSA	50	1	Bajrayogini	Rhesus	**POS**	**POS**	**III**	**8**
UNKNOWN 12	ST(4-421-1-105-1-5-x)-MSSA	61	1	Hetauda	Rhesus	**POS**	**POS**	**IV**	**5**
UNKNOWN 13	ST(1-3-1-x-1-1-11)-MSSA	62, 72	2	1 Chitwan, 1 Hetauda	Rhesus	**POS**	**POS**	**IV**	**5**
UNKNOWN 14	ST(3-38-1-15-1-x-40)-MSSA	G	1	Guheswari	Rhesus	**POS**	**POS**	**IV**	**5**
UNKNOWN 15	ST(1-421-1-1-12-1-11)-MSSA	11, E	2	1 Gokarna, 1 Thapathali	Rhesus	**POS**	**POS**	**IV**	**5**
UNKNOWN 16	ST(3-3-1-66-4-x-x)-MSSA	49, I	2	1 Bajrayogini, 1 Guheswari	Rhesus	**POS**	**POS**	**IV**	**8**
UNKNOWN 17	MSSA	13	1	Thapathali	Rhesus	**POS**	**POS**	**IV**	**8**

**Table 2:** Susceptibility tests.

Clonal Complex	Strain	Number	PEN	OXA	FOX	GEN	TOB	CIP	LEV	MOX	ERY	CLI	LIN	TEI	VAN	TET	FOS	NIF	FUS	MUP	RIF	TSU
**CC06**	CC6-MRSA-IV	1	**R**	**R**	**R**	**S**	**S**	**S**	**S**	**S**	**R**	**R ^a^**	S	S	S	S	S	S	S	S	S	S
**CC22**	CC22-MRSA-IV (PVL+/*tst*+)	5	**R**	**R**	**R**	**R**	**R**	**R**	**R**	**R**	**VAR ^b^**	**VAR ^ab^**	S	S	S	S	S	S	S	S	S	**VAR ^c^**
**CC772**	CC772-MRSA-V (PVL+), “Bengal Bay”	1	**R**	**R**	**R**	**R**	**R**	**R**	**R**	**R**	**I**	S	S	S	S	S	S	I	S	S	S	**R**
**CC15**	CC15-MSSA	1	**R**	S	S	S	S	**R**	**R**	**R**	**R**	**R ^a^**	S	S	S	S	S	S	S	S	S	S
**CC96**	CC96-MSSA	1	S	S	S	S	S	S	S	S	**R**	S	S	S	S	S	S	S	S	S	S	S
**CC2990**	CC2990-MSSA	1	S	S	S	S	S	S	S	S	S	S	S	S	S	S	S	S	S	S	S	S
**UNKNOWN 01**	ST(4-13-1-105-11-5-x)-MSSA	2	S	S	S	S	S	S	S	S	S	S	S	S	S	S	S	I	S	S	S	S
**UNKNOWN 02**	ST(1-421-1-1-12-238-11)-MSSA	2	S	S	S	S	S	S	S	S	S	S	S	S	S	S	S	S	S	S	S	S
**UNKNOWN 03**	ST(1-3-1-15-28-x-1)-MSSA	4	S	S	S	S	S	S	S	S	S	S	S	S	S	S	S	S	S	S	S	S
**UNKNOWN 04**	MSSA	1	S	S	S	S	S	S	S	S	S	S	S	S	S	S	S	S	S	S	S	S
**UNKNOWN 05**	ST(1-1-1-1-28-4-11)-MSSA	2	S	S	S	S	S	S	S	S	S	S	S	S	S	S	S	S	S	S	S	S
**UNKNOWN 06**	ST(1-421-1-x-x-1-11)-MSSA	1	S	S	S	S	S	S	S	S	S	S	S	S	S	S	S	S	S	S	S	S
**UNKNOWN 07**	ST(12-x-1-66-11-x-x)-MSSA	3	S	S	S	S	S	S	S	S	S	S	S	S	S	S	S	S	S	S	S	S
**UNKNOWN 08**	ST(1-38-1-1-x-238-x)-MSSA	2	S	S	S	S	S	S	S	S	S	S	S	S	S	S	S	I	S	S	S	S
**UNKNOWN 09**	ST(3-3-1-66-28-x-x)-MSSA	1	S	S	S	S	S	S	S	S	S	S	S	S	S	S	S	S	S	S	S	S
**UNKNOWN 10**	ST(3-1-1-66-28-1-x)-MSSA	2	S	S	S	S	S	S	S	S	S	S	S	S	S	S	S	S	S	S	S	S
**UNKNOWN 11**	MSSA	1	S	S	S	S	S	S	S	S	S	S	S	S	S	S	S	S	S	S	S	S
**UNKNOWN 12**	ST(4-421-1-105-1-5-x)-MSSA	1	S	S	S	S	S	S	S	S	S	S	S	S	S	S	S	S	S	S	S	S
**UNKNOWN 13**	ST(1-3-1-x-1-1-11)-MSSA	2	S	S	S	S	S	S	S	S	S	S	S	S	S	S	S	I	S	S	S	S
**UNKNOWN 14**	ST(3-38-1-15-1-x-40)-MSSA	1	S	S	S	S	S	S	S	S	S	S	S	S	S	S	S	S	S	S	S	S
**UNKNOWN 15**	ST(1-421-1-1-12-1-11)-MSSA	2	S	S	S	S	S	S	S	S	S	S	S	S	S	S	S	S	S	S	S	S
**UNKNOWN 16**	ST(3-3-1-66-4-1-x)-MSSA	2	S	S	S	S	S	S	S	S	S	S	S	S	S	S	S	S	S	S	S	S
**UNKNOWN 17**	MSSA	1	S	S	S	S	S	S	S	S	S	S	S	S	S	S	S	S	S	S	S	S

PEN, Benzylpenicillin; OXA, Oxacillin; FOX, Cefoxitin; GEN, Gentamicin; TOB, Tobramycin; CIP, Ciprofloxacin; LEV, Levofloxacin; MOX, Moxifloxacin; ERY, Erythromycin; CLI, Clindamycin; LIN, Linezolid; TEI, Teicoplanin; VAN, Vancomycin; TET, Tetracycline; FOS, Fosfomycin; NIF, Nitrofurantoin; FUS, Fusidic acid; MUP, Mupirocin; RIF, Rifampicin and TSU, Trimethoprim/Sulfamethoxazole ^a^ Inducible clindamycin resistance. ^b^ Variable, four out of five isolates resistant (ERY)/inducibly resistant (CLI); one susceptible. ^c^ Variable, three out of five isolates resistant, two susceptible. The actual MICs are found in Supplementary Table S2.

**Table 3:** SCCmec markers and resistance genes ^a^.

Clonal Complex	Strain	Number	*mecA*	Delta *mecR*	*ccrA-2*	*ccrB-2*	*ccrAA*	*ccrC*	*blaZ/I/R*	*erm*(C)	*lnu*(A)	*msr*(A)	*mpB*(C)	*aacA-aphD*	*aphA3+sat*	*dfrA*
**CC06**	CC6-MRSA-IV	1	**POS**	**POS**	**POS**	**POS**	NEG	NEG	NEG	**POS**	NEG	NEG	NEG	NEG	NEG	NEG
**CC22**	CC22-MRSA-IV (PVL+/*tst*+)	5	**POS**	**POS**	**POS**	**POS**	NEG	NEG	**POS**	**VAR ^b^**	NEG	NEG	NEG	**POS**	NEG	**POS**
**CC772**	CC772-MRSA-V(PVL+), “Bengal Bay “	1	**POS**	NEG	NEG	NEG	**POS**	**POS**	**POS**	NEG	NEG	**POS**	**POS**	**POS**	**POS**	NEG
**CC15**	CC15-MSSA	1	NEG	NEG	NEG	NEG	NEG	NEG	**POS**	**POS**	NEG	NEG	NEG	NEG	NEG	NEG
**CC96**	CC96-MSSA	1	NEG	NEG	NEG	NEG	NEG	NEG	NEG	NEG	NEG	**POS**	NEG	NEG	NEG	NEG
**CC2990**	CC2990-MSSA	1	NEG	NEG	NEG	NEG	NEG	NEG	NEG	NEG	NEG	NEG	NEG	NEG	NEG	NEG
**UNKNOWN 01**	ST(4-13-1-105-11-5-x)-MSSA	2	NEG	NEG	NEG	NEG	NEG	NEG	NEG	NEG	NEG	NEG	NEG	NEG	NEG	NEG
**UNKNOWN 02**	ST(1-421-1-1-12-238-11)-MSSA	2	NEG	NEG	NEG	NEG	NEG	NEG	NEG	NEG	NEG	NEG	NEG	NEG	NEG	NEG
**UNKNOWN 03**	ST(1-3-1-15-28-x-1)-MSSA	4	NEG	NEG	NEG	NEG	NEG	NEG	NEG	NEG	NEG	NEG	NEG	NEG	NEG	NEG
**UNKNOWN 04**	MSSA	1	NEG	NEG	NEG	NEG	NEG	NEG	NEG	NEG	NEG	NEG	NEG	NEG	NEG	NEG
**UNKNOWN 05**	ST(1-1-1-1-28-4-11)-MSSA	2	NEG	NEG	NEG	NEG	NEG	NEG	NEG	NEG	NEG	NEG	NEG	NEG	NEG	NEG
**UNKNOWN 06**	ST(1-421-1-x-x-1-11)-MSSA	1	NEG	NEG	NEG	NEG	NEG	NEG	NEG	NEG	NEG	NEG	NEG	NEG	NEG	NEG
**UNKNOWN 07**	ST(12-x-1-66-11-x-x)-MSSA	3	NEG	NEG	NEG	NEG	NEG	NEG	NEG	NEG	NEG	NEG	NEG	NEG	NEG	NEG
**UNKNOWN 08**	ST(1-38-1-1-x-238-x)-MSSA	2	NEG	NEG	NEG	NEG	NEG	NEG	NEG	NEG	NEG	NEG	NEG	NEG	NEG	NEG
**UNKNOWN 09**	MSSA	1	NEG	NEG	NEG	NEG	NEG	NEG	NEG	NEG	NEG	NEG	NEG	NEG	NEG	NEG
**UNKNOWN 10**	ST(3-3-1-66-28-x-x)-MSSA	2	NEG	NEG	NEG	NEG	NEG	NEG	NEG	NEG	NEG	NEG	NEG	NEG	NEG	NEG
**UNKNOWN 11**	MSSA	1	NEG	NEG	NEG	NEG	NEG	NEG	NEG	NEG	NEG	NEG	NEG	NEG	NEG	NEG
**UNKNOWN 12**	ST(4-421-1-105-1-5-x)-MSSA	1	NEG	NEG	NEG	NEG	NEG	NEG	NEG	NEG	NEG	NEG	NEG	NEG	NEG	NEG
**UNKNOWN 13**	ST(1-3-1-x-1-1-11)-MSSA	2	NEG	NEG	NEG	NEG	NEG	NEG	NEG	NEG	NEG	NEG	NEG	NEG	NEG	NEG
**UNKNOWN 14**	ST(3-38-1-15-1-x-40)-MSSA	1	NEG	NEG	NEG	NEG	NEG	NEG	NEG	NEG	NEG	NEG	NEG	NEG	NEG	NEG
**UNKNOWN 15**	ST(1-421-1-1-12-1-11)-MSSA	2	NEG	NEG	NEG	NEG	NEG	NEG	NEG	NEG	NEG	NEG	NEG	NEG	NEG	NEG
**UNKNOWN 16**	ST(3-3-1-66-4-1-x)-MSSA	2	NEG	NEG	NEG	NEG	NEG	NEG	NEG	NEG	NEG	NEG	NEG	NEG	NEG	NEG
**UNKNOWN 17**	MSSA	1	NEG	NEG	NEG	NEG	NEG	NEG	NEG	NEG	NEG	NEG	NEG	NEG	NEG	NEG

^a^ The table shows only genes that were found at least once in this study. Genes that were *not* present in any of the study strains are other ccrA/B genes, *mecR*, *mecI*, *kdp*-operon genes, heavy metal resistance genes, *mecC*, *blaZ* SCC*mec* XI, *erm*(A),* erm*(B),* lnu*(A),* mef*(A),* vat*(A),* vat*(B),* vga*(A),* vga*(A),* vgb*(A),* far1*, *fusC*, *mupR*, *tet*(K),* tet*(M),* cat*, *cfr*, *fexA*, *qacA*, *qacC*, *vanA*, *vanB*, and *vanZ*. ^b^ Variable, present in four out of five isolates.

**Table 4:** Virulence-associated genes ^a^.

Clonal Complex	Strain	Number	*tst*	sea	*sec+sel*	*egc* Genes	ORF CM14	PVL	*lukD/E* ^b^	*sak*	*chp*	*scn*	*edinB+etD2*	*cna*	*sasG*
**CC06**	CC6-MRSA-IV	1	NEG	NEG	NEG	NEG	NEG	NEG	**POS**	**POS**	NEG	**POS**	NEG	**POS**	**POS**
**CC22**	CC22-MRSA-IV (PVL+/*tst*+)	5	**POS**	NEG	**POS**	**POS**	NEG	**POS**	NEG	**POS**	**POS**	**POS**	NEG	**POS**	**POS**
**CC772**	CC772-MRSA-V (PVL+), “Bengal Bay “	1	NEG	**POS**	**POS**	**POS**	**POS**	**POS**	NEG	NEG	NEG	**POS**	NEG	**POS**	**POS**
**CC15**	CC15-MSSA	1	NEG	NEG	NEG	NEG	NEG	NEG	**POS**	NEG	**POS**	**POS**	NEG	NEG	**POS**
**CC96**	CC96-MSSA	1	NEG	NEG	NEG	NEG	NEG	NEG	**POS**	NEG	NEG	NEG	NEG	**POS**	**POS**
**CC2990**	CC2990-MSSA	1	NEG	NEG	POS	NEG	NEG	NEG	**(POS) ^b^**	**POS**	**POS**	**POS**	**POS**	**POS**	**POS**
**UNKNOWN 01**	ST(4-13-1-105-11-5-x)-MSSA	2	NEG	NEG	NEG	NEG	NEG	NEG	**POS**	NEG	NEG	NEG	**POS**	NEG	**POS**
**UNKNOWN 02**	ST(1-421-1-1-12-238-11)-MSSA	2	NEG	NEG	NEG	POS	NEG	NEG	**POS**	NEG	NEG	NEG	NEG	**POS**	**POS**
**UNKNOWN 03**	ST(1-3-1-15-28-x-1)-MSSA	4	NEG	NEG	NEG	NEG	NEG	NEG	**POS**	NEG	NEG	NEG	NEG	**POS**	NEG
**UNKNOWN 04**	MSSA	1	NEG	NEG	NEG	NEG	NEG	NEG	**(POS) ^b^**	NEG	NEG	NEG	NEG	NEG	NEG
**UNKNOWN 05**	ST(1-1-1-1-28-4-11)-MSSA	2	NEG	NEG	NEG	NEG	NEG	NEG	**POS**	NEG	NEG	NEG	**POS**	**POS**	**POS**
**UNKNOWN 06**	ST(1-421-1-x-x-1-11)-MSSA	1	NEG	NEG	NEG	POS	NEG	NEG	**POS**	NEG	NEG	NEG	NEG	**POS**	NEG
**UNKNOWN 07**	ST(12-x-1-66-11-x-x)-MSSA	3	NEG	NEG	NEG	NEG	NEG	NEG	**POS**	NEG	NEG	NEG	NEG	NEG	NEG
**UNKNOWN 08**	ST(1-38-1-1-x-238-x)-MSSA	2	NEG	NEG	NEG	NEG	NEG	NEG	**POS**	NEG	NEG	NEG	NEG	**POS**	NEG
**UNKNOWN 09**	ST(3-3-1-66-28-x-x)-MSSA	1	NEG	NEG	NEG	NEG	NEG	NEG	**(POS) ^b^**	NEG	NEG	NEG	NEG	NEG	NEG
**UNKNOWN 10**	ST(3-1-1-66-28-1-x)-MSSA	2	NEG	NEG	NEG	NEG	NEG	NEG	**POS**	NEG	NEG	NEG	NEG	NEG	**POS**
**UNKNOWN 11**	MSSA	1	NEG	NEG	NEG	NEG	NEG	NEG	**POS**	NEG	NEG	NEG	NEG	NEG	**POS**
**UNKNOWN 12**	ST(4-421-1-105-1-5-x)-MSSA	1	NEG	NEG	NEG	NEG	NEG	NEG	**POS**	NEG	NEG	NEG	NEG	NEG	**POS**
**UNKNOWN 13**	ST(1-3-1-x-1-1-11)-MSSA	2	NEG	NEG	NEG	NEG	NEG	NEG	**POS**	NEG	NEG	NEG	**POS**	NEG	**VAR ^c^**
**UNKNOWN 14**	ST(3-38-1-15-1-x-40)-MSSA	1	NEG	NEG	NEG	NEG	NEG	NEG	**POS**	NEG	NEG	NEG	**POS**	**POS**	**POS**
**UNKNOWN 15**	ST(1-421-1-1-12-1-11)-MSSA	2	NEG	NEG	NEG	POS	NEG	NEG	**POS**	NEG	NEG	NEG	NEG	NEG	**POS**
**UNKNOWN 16**	ST(3-3-1-66-4-1-x)-MSSA	2	NEG	NEG	NEG	NEG	NEG	NEG	**POS**	NEG	NEG	NEG	NEG	NEG	NEG
**UNKNOWN 17**	MSSA	1	NEG	NEG	NEG	POS	NEG	NEG	**POS**	NEG	NEG	NEG	NEG	**POS**	NEG

^a^ Genes that were not present in any of the study strains are *seb*, *sed*, *sej*, *ser*, *seh*, *see*, *lukF-*P83+*lukM*, *edinA*, *edinC*, *eta*, *etb*, and *etD*. Genes present in *all* strains were *hla*, *hld*, *lukF/S-hlg*, *lukX/Y*, *aur*, and *icaA+C+D*. See Supplemental File for more details. ^b^ (POS) indicates that *lukD* was detected by array, while the *lukE* probe yielded no signals. This is more likely due to allelic variation rather than due to true absence. ^c^ Variable, present in one out of two isolates.

## Supplementary Materials

The following are available online at https://www.mdpi.com/2079-6382/9/10/689/s1, Table S1: MLST Sequences. Table S2 MICs of the Isolates.
