# Supplementary material for: Staphylococcus aureus and Methicillin Resistant S. aureus in Nepalese Primates: Resistance to Antimicrobials, Virulence, and Genetic Lineages
Source: Antibiotics (Basel). 2020 Oct 13;9(10):689. doi: 10.3390/antibiotics9100689 (PMC7601186; doi:10.3390/antibiotics9100689)
Supplement: Supplementary file 1 [file antibiotics-09-00689-s001.zip › antibiotics-946806 supple/Nepalese Monkey strains_SUPPLEMENTAL FILE _2020-09-30.docx]

**Supplemental file 1:** MLST Sequences.

| **Isolate** | **MLST Allele** | **nt** | **Sequence** |
| --- | --- | --- | --- |
| Guheswari-H (ST2990) | arcC-0001 | 456 | TTATTAATCCAACAAGCTAAATCGAACAGTGACACAACGCCGGCAATGCCATTGGATACTTGTGGTGCAATGTCACAGGGTATGATAGGCTATTGGTTGGAAACTGAAATCAATCGCATTTTAACTGAAATGAATAGTGATAGAACTGTAGGCACAATCGTTACACGTGTGGAAGTAGATAAAGATGATCCACGATTCAATAACCCAACCAAACCAATTGGTCCTTTTTATACGAAAGAAGAAGTTGAAGAATTACAAAAAGAACAGCCAGACTCAGTCTTTAAAGAAGATGCAGGACGTGGTTATAGAAAAGTAGTTGCGTCACCACTACCTCAATCTATACTAGAACACCAGTTAATTCGAACTTTAGCAGACGGTAAAAATATTGTCATTGCATGCGGTGGTGGCGGTATTCCAGTTATAAAAAAAGAAAATACCTATGAAGGTGTTGAAGCG |
|  | aroE-0001 | 456 | AATTTTAATTCTTTAGGATTAGATGATACTTATGAAGCTTTAAATATTCCAATTGAAGATTTTCATTTAATTAAAGAAATTATTTCGAAAAAAGAATTAGATGGCTTTAATATCACAATTCCTCATAAAGAACGTATCATACCGTATTTAGATCATGTTGATGAACAAGCGATTAATGCAGGTGCAGTTAACACTGTTTTGATAAAAGATGACAAGTGGATAGGGTATAATACAGATGGTATTGGTTATGTTAAAGGATTGCACAGCGTTTATCCAGATTTAGAAAATGCATACATTTTAATTTTGGGCGCAGGTGGTGCAAGTAAAGGTATTGCTTATGAATTAGCAAAATTTGTAAAGCCCAAATTAACTGTTGCGAATAGAACGATGGCTCGTTTTGAATCTTGGAATTTAAATATAAACCAAATTTCATTAGCAGATGCTGAAAAGTATTTA |
|  | glpF-0001 | 465 | GGTGCTGATTGGATTGTCATCACAGCTGGATGGGGATTAGCGGTTACAATGGGTGTGTTTGCTGTCGGTCAATTCTCAGGTGCACATTTAAACCCAGCGGTGTCTTTAGCTCTTGCATTAGACGGAAGTTTTGATTGGTCATTAGTTCCTGGTTATATTGTTGCTCAAATGTTAGGTGCAATTGTCGGAGCAACAATTGTATGGTTAATGTACTTGCCACATTGGAAAGCGACAGAAGAAGCTGGCGCGAAATTAGGTGTTTTCTCTACAGCACCGGCTATTAAGAATTACTTTGCCAACTTTTTAAGTGAGATTATCGGAACAATGGCATTAACTTTAGGTATTTTATTTATCGGTGTAAACAAAATTGCCGATGGTTTAAATCCTTTAATTGTCGGAGCATTAATTGTTGCAATCGGATTAAGTTTAGGCGGTGCTACTGGTTATGCAATCAACCCAGCACGT |
|  | gmk-0001 | 417 | CGAATATTTGAAGATCCAAGTACATCATATAAGTATTCTATTTCAATGACAACACGTCAAATGCGTGAAGGTGAAGTTGATGGCGTAGATTACTTTTTTAAAACTAGGGATGCGTTTGAAGCTTTAATCAAAGATGACCAATTTATAGAATATGCTGAATATGTAGGCAACTATTATGGTACACCAGTTCAATATGTTAAAGATACAATGGACGAAGGTCATGATGTATTTTTAGAAATTGAAGTAGAAGGTGCAAAGCAAGTTAGAAAGAAATTTCCAGATGCGCTATTTATTTTCTTAGCACCTCCAAGTTTAGAACACTTGAGAGAGCGATTAGTAGGTAGAGGAACAGAATCTGATGAGAAAATACAAAGTCGTATTAACGAAGCGCGTAAAGAAGTTGAAATGATGAATTTA |
|  | pta-0330 | 474 | GCAACACAATTACAAGCAACAGATTATGTTACACCAATCGTGTTAGGTGATGAGACTAAGGTTCAATCTTTAGCGCAAAAACTTGATCTTGATATTTCTAATATTGAATTAATTAATCCTGCGACAAGTGAATTGAAAGCTGAATTAGTTCAATCATTTGTTGAACGACGTAAAGGTAAAGCGACTGAAGAACAAGCACAAGAATTATTAAACAATGTGAACTACTTCGGTACAATGCTTGTTTATGCTGGTAAAGCAGATGGTTTAGTTAGTGGTGCAGCACATTCAACAGGAGACACTGTGCGTCCAGCTTTACAAATCATCAAAACTAAACCAGGTGTATCAAGAACATCAGGTATCTTCTTTATGATTAAAGGTGATGAACAATACATCTTTGGTGATTGTGCAATCAATCCAGAACTTGATTCACAAGGACTTGCAGAAATTGCAGTAGAAAGTGCAAAATCAGCATTA |
|  | tpi-0001 | 402 | CACGAAACAGATGAAGAAATTAACAAAAAAGCGCACGCTATTTTCAAACATGGAATGACTCCAATTATTTGTGTTGGTGAAACAGACGAAGAGCGTGAAAGTGGTAAAGCTAACGATGTTGTAGGTGAGCAAGTTAAGAAAGCTGTTGCAGGTTTATCTGAAGATCAACTTAAATCAGTTGTAATTGCTTATGAGCCAATCTGGGCAATCGGAACTGGTAAATCATCAACATCTGAAGATGCAAATGAAATGTGTGCATTTGTACGTCAAACTATTGCTGACTTATCAAGCAAAGAAGTATCAGAAGCAACTCGTATTCAATATGGTGGTAGTGTTAAACCTAACAACATTAAAGAATACATGGCACAAACTGATATTGATGGGGCATTAGTAGGTGGCGCA |
|  | yqiL-0010 | 516 | GCGTTTAAAGACGTGCCAGCCTATGATTTAGGTGCGACTTTAATAGAACATATTATTAAAGAGACGGGTTTGAATCCAAGTGAGATTGATGAAGTTATCATCGGTAACGTACTACAAGCAGGACAAGGACAAAATCCAGCACGAATTGCTGCTATGAAAGGTGGCTTGCCAGAAACAGTACCTGCATTTACAGTGAATAAAGTATGTGGTTCTGGGTTAAAGTCGATTCAATTAGCATATCAATCTATTGTGACTGGTGAAAATGACATCGTGCTAGCTGGCGGTATGGAGAATATGTCTCAGTCACCAATGCTTGTCAACAACAGTCGCTTCGGTTTTAAAATGGGACATCAATCAATGGTTGATAGCATGGTATATGATGGTTTAACAGATGTATTTAATCAATATCATATGGGTATTACTGCTGAAAATTTAGTAGAGCAATATGGTATTTCAAGAGAAGAACAAGATACATTTGCTGTAAACTCACAACATAAAGCAGTACGTGCACAGCAA |

| **Isolate** | **MLST Allele** | **nt** | **Sequence** |
| --- | --- | --- | --- |
| Ramdi-86 ("UNKNOWN 01") | arcC-0004 | 456 | TTATTAATCCAACAAGCTAAATCGAACAGTGACACAACGCCGGCAATGCCATTGGATACTTGTGGTGCAATGTCACAGGGTATGATAGGCTATTGGTTGGAAACTGAAATCAATCGCATTTTAACTGAAATGAATAGTGATAGAACCGTAGGCACAATCGTTACACGTGTGGAAGTAGATAAAGATGATCCACGATTCAATAACCCAACCAAACCAATTGGTCCTTTTTATACGAAAGAAGAAGTTGAAGAATTACAAAAAGAACAGCCAGACTCAGTCTTTAAAGAAGATGCAGGACGTGGTTATAGAAAAGTAGTTGCGTCACCACTACCTCAATCTATACTAGAACACCAGTTAATTCGAACTTTAGCAGACGGAAAAAATATTGTCATTGCATGCGGTGGTGGCGGTATTCCAGTTATAAAAAAAGAAAATACCTATGAAGGTGTTGAAGCG |
|  | aroE-0013 | 456 | AATTTTAATTCTTTAGGATTAGCTGATACTTATGAAGCTTTAAATATTCCAATTGAAGATTTTCATTTAATTAAAGAAATTATTTCAAAAAAAGAATTAGATGGCTTTAATATCACAATTCCTCATAAAGAACGTATCATATCGTATTTAGATCATGTTGATGAACAAGCGATTAATGCAGGTGCAGTTAACACTGTTTTGATAAAAGATGGCAAGTGGATAGGGTATAATACAGATGGTATTGGTTATGTTAAAGGATTGCACAGCGTTTATCCAGATTTAGAAAATGCATACATTTTAATTTTGGGCGCAGGTGGTGCAAGTAAAGGCATTGCTTATGAATTAGCAAAATTTGTAAAGCCCAAATTAACTGTTGCGAATAGAACGATGGCTCGTTTTGAATCTTGGAATTTAAATATAAACCAAATTTCATTAGCAGATGCTGAAAAGTATTTA |
|  | glpF-0001 | 465 | GGTGCTGATTGGATTGTCATCACAGCTGGATGGGGATTAGCGGTTACAATGGGTGTGTTTGCTGTCGGTCAATTCTCAGGTGCACATTTAAACCCAGCGGTGTCTTTAGCTCTTGCATTAGACGGAAGTTTTGATTGGTCATTAGTTCCTGGTTATATTGTTGCTCAAATGTTAGGTGCAATTGTCGGAGCAACAATTGTATGGTTAATGTACTTGCCACATTGGAAAGCGACAGAAGAAGCTGGCGCGAAATTAGGTGTTTTCTCTACAGCACCGGCTATTAAGAATTACTTTGCCAACTTTTTAAGTGAGATTATCGGAACAATGGCATTAACTTTAGGTATTTTATTTATCGGTGTAAACAAAATTGCCGATGGTTTAAATCCTTTAATTGTCGGAGCATTAATTGTTGCAATCGGATTAAGTTTAGGCGGTGCTACTGGTTATGCAATCAACCCAGCACGT |
|  | gmk-0105 | 417 | CGAATATTTGAAGATCCAAGTACATCATATAAGTATTCTATTTCAATGACAACACGTCAAATGCGTGAAGGTGAAGTTGATGGCGTAGATTACTTTTTTAAAACTAGGGATGCGTTTGAAGCTTTAATCAAAGATGACCAATTTATAGAATATGCTGAATATGTAGGCAACTATTATGGTACACCAGTTCAATATGTTAAAGATACAATGGACGAAGGTCATGATGTATTTTTAGAAATTGAAGTAGAAGGTGCAAAGCAAGTTAGAAAGAAATTTCCAGATGCGTTATTTATTTTCTTAGCACCTCCAAGTTTAGATCACTTGAGAGAGCGATTAGTAGGTAGAGGAACAGAATCCGATGAGAAAATACAAAGTCGTATTAACGAAGCGCGTAAAGAAGTTGAAATGATGAATTTA |
|  | pta-0011 | 474 | GCAACACAATTACAAGCAACAGATTATGTTACACCAATCGTGTTAGGTGATGAGACTAAGGTTCAATCTTTAGCGCAAAAACTTAATCTTGATATTTCTAATATTGAATTAATTAATCCTGCGACAAGTGAATTGAAAGCTGAATTAGTCCAATCATTTGTTGAACGACGTAAAGGTAAAGCGACTGAAGAACAAGCGCAAGAATTATTAAACAATGTGAACTACTTCGGTACAATGCTTGTTTATGCTGGTAAAGCAGATGGTTTAGTTAGTGGTGCAGCACATTCAACAGGCGACACTGTGCGTCCAGCTTTACAAATCATCAAAACGAAACCAGGTGTATCAAGAACATCAGGTATCTTCTTTATGATTAAAGGTGATGAACAATACATCTTTGGTGATTGTGCAATCAATCCAGAACTTGATTCACAAGGACTTGCAGAAATTGCAGTAGAAAGTGCAAAATCAGCATTA |
|  | tpi-0005 | 402 | CACGAAACAGATGAAGAAATTAACAAAAAAGCGCACGCTATTTTCAAACATGGAATGACTCCAATTATATGTGTTGGTGAAACAGACGAAGAGCGTGAAAGTGGTAAAGCTAACGATGTTGTAGGTGAGCAAGTTAAGAAAGCTGTTGCAGGTTTATCTGAAAATCAACTAAAATCAGTTGTAATTGCTTATGAACCAATCTGGGCAATCGGAACTGGTAAATCATCAACATCTGAAGATGCGAATGAAATGTGTGCATTTGTACGTCAAACTATTGCTGACTTATCAAGCAAAGAAGTATCAGAAGCAACTCGTATTCAATATGGTGGTAGTGTTAAACCTAACAACATTAAAGAATACATGGCACAAACTGATATTGATGGGGCATTAGTAGGTGGCGCA |
|  | yqiL – un-known allele | 516 | GCGTTTAAAGACGTGCCAGCCTATGATTTAGGTGCGACTTTAATAGAACATATTATTAAAGAGACGGGTTTGAATCCTAGTGAGATTGATGAAGTTATCATCGGTAACGTACTACAAGCAGGACAAGGACAAAATCCAGCACGAATTGCTGCTATGAAAGGTGGCTTGCCAGAAACAGTACCTGCATTTACGGTGAATAAAGTATGTGGTTCTGGGTTAAAGTCGATTCAATTAGCATATCAATCTATTGTGACTGGTGAAAATGACATCGTGCTAGCTGGCGGTATGGAGAATATGTCTCAGTCACCAATGCTTGTCAACAACAGTCGCTTCGGTTTTAAAATGGGACATCAATCAATGGTTGATAGCATGGTATATGATGGTTTAACAGATGTATTTAATCAATATCATATGGGTATTACTGCTGAAAATTTAGTAGAGCAATATGGTATTTCAAGAGAAGAACAAGATACATTTGCTGTAAACTCACAACAAAAAGCAGTACGTGCACAGCAA |

| **Isolate** | **MLST Allele** | **nt** | **Sequence** |
| --- | --- | --- | --- |
| Gokarna-52 ("UNKNOWN 02") | arcC-0001 | 456 | TTATTAATCCAACAAGCTAAATCGAACAGTGACACAACGCCGGCAATGCCATTGGATACTTGTGGTGCAATGTCACAGGGTATGATAGGCTATTGGTTGGAAACTGAAATCAATCGCATTTTAACTGAAATGAATAGTGATAGAACTGTAGGCACAATCGTTACACGTGTGGAAGTAGATAAAGATGATCCACGATTCAATAACCCAACCAAACCAATTGGTCCTTTTTATACGAAAGAAGAAGTTGAAGAATTACAAAAAGAACAGCCAGACTCAGTCTTTAAAGAAGATGCAGGACGTGGTTATAGAAAAGTAGTTGCGTCACCACTACCTCAATCTATACTAGAACACCAGTTAATTCGAACTTTAGCAGACGGTAAAAATATTGTCATTGCATGCGGTGGTGGCGGTATTCCAGTTATAAAAAAAGAAAATACCTATGAAGGTGTTGAAGCG |
|  | aroE-0421 | 456 | AATTTTAATTCTTTAGGATTAGATGATACTTATGAAGCTTTAAATATTCCAATTGAAGATTTTCATTTAATTAAAGAAATTATTTCAAAAAAAGAATTAGATGGCTTTAATATCACAATTCCTCATAAAGAACGTATCATATCGTATTTAGATCATGTTGATGAACAAGCGATTAATGCAGGTGCAGTTAACACTGTTTTGATAAAAGATGGCAAGTGGATAGGGTATAATACAGATGGTATTGGTTATGTTAAAGGATTGCACAGCGTTTATCCAGATTTAGAAAATGCATACATTTTAATTTTGGGCGCAGGTGGTGCAAGTAAAGGCATTGCTTATGAATTAGCAAAATTTGTAAAGCCCAAATTAACTGTTGCGAATAGAACGATGGCTCGTTTTGAATCTTGGAATTTAAATATAAACCAAATTTCATTAGCAGATGCTGAAAAGTATTTA |
|  | glpF-0001 | 465 | GGTGCTGATTGGATTGTCATCACAGCTGGATGGGGATTAGCGGTTACAATGGGTGTGTTTGCTGTCGGTCAATTCTCAGGTGCACATTTAAACCCAGCGGTGTCTTTAGCTCTTGCATTAGACGGAAGTTTTGATTGGTCATTAGTTCCTGGTTATATTGTTGCTCAAATGTTAGGTGCAATTGTCGGAGCAACAATTGTATGGTTAATGTACTTGCCACATTGGAAAGCGACAGAAGAAGCTGGCGCGAAATTAGGTGTTTTCTCTACAGCACCGGCTATTAAGAATTACTTTGCCAACTTTTTAAGTGAGATTATCGGAACAATGGCATTAACTTTAGGTATTTTATTTATCGGTGTAAACAAAATTGCCGATGGTTTAAATCCTTTAATTGTCGGAGCATTAATTGTTGCAATCGGATTAAGTTTAGGCGGTGCTACTGGTTATGCAATCAACCCAGCACGT |
|  | gmk-0001 | 417 | CGAATATTTGAAGATCCAAGTACATCATATAAGTATTCTATTTCAATGACAACACGTCAAATGCGTGAAGGTGAAGTTGATGGCGTAGATTACTTTTTTAAAACTAGGGATGCGTTTGAAGCTTTAATCAAAGATGACCAATTTATAGAATATGCTGAATATGTAGGCAACTATTATGGTACACCAGTTCAATATGTTAAAGATACAATGGACGAAGGTCATGATGTATTTTTAGAAATTGAAGTAGAAGGTGCAAAGCAAGTTAGAAAGAAATTTCCAGATGCGCTATTTATTTTCTTAGCACCTCCAAGTTTAGAACACTTGAGAGAGCGATTAGTAGGTAGAGGAACAGAATCTGATGAGAAAATACAAAGTCGTATTAACGAAGCGCGTAAAGAAGTTGAAATGATGAATTTA |
|  | pta-0012 | 474 | GCAACACAATTACAAGCAACAGATTATGTTACACCAATCGTGTTAGGTGATGAGACTAAGGTTCAATCTTTAGCGCAAAAACTTGATCTTGATATTTCTAATATTGAATTAATTAATCCTGCGACAAGTGAATTGAAAGCTGAATTAGTTCAATCATTTGTTGAACGACGTAAAGGTAAAGCGACTGAAGAACAAGCACAAGAATTATTAAACAATGTGAACTACTTCGGTACAATGCTTGTTTATGCTGGTAAAGCAGATGGTTTAGTTAGTGGTGCAGCACATTCAACAGGAGACACTGTGCGTCCAGCTTTACAAATCATCAAAACGAAACCAGGTGTATCAAGAACATCAGGTATCTTCTTTATGATTAAAGGTGATGAACAATACATCTTTGGTGATTGTGCAATCAATCCAGAACTTGATTCACAAGGACTTGCAGAAATTGCAGTAGAAAGTGCAAAATCAGCATTA |
|  | tpi-0238 | 402 | CACGAAACAGATGAAGAAATTAACAAAAAAGCGCACGCTATTTTCAAACATGGAATGACGCCAATTATTTGTGTTGGTGAAACAGACGAAGAGCGTGAAAGTGGTAAAGCTAACGATGTTGTAGGTGAGCAAGTTAAGAAAGCTGTTGCAGGTTTATCTGAAGATCAACTTAAATCAGTTGTAATTGCTTATGAACCAATCTGGGCAATCGGAACTGGTAAATCATCAACATCTGAAGATGCAAATGAAATGTGTGCATTTGTACGTCAAACTATTGCTGACTTATCAAGCAAAGAAGTATCAGAAGCAACTCGTATTCAATATGGTGGTAGTGTTAAACCTAACAACATTAAAGAATACATGGCACAAACTGATATTGATGGGGCATTAGTAGGTGGCGCA |
|  | yqiL-0011 | 516 | GCGTTTAAAGACGTGCCAGCCTATGATTTAGGTGCGACTTTAATAGAACATATTATTAAAGAGACGGGTTTGAATCCAAGTGAGATTGATGAAGTTATCATCGGTAACGTACTACAAGCAGGACAAGGACAAAATCCAGCACGAATTGCTGCTATGAAAGGTGGCTTGCCAGAAACAGTACCTGCATTTACAGTGAATAAAGTATGTGGTTCTGGGTTAAAGTCGATTCAATTAGCATATCAATCTATTGTGACTGGTGAAAATGACATCGTGCTAGCTGGCGGTATGGAGAATATGTCTCAATCACCAATGCTTGTCAACAACAGTCGCTTTGGTTTTAAAATGGGACATCAATCAATGGTTGATAGCATGGTATATGATGGTTTAACAGATGTATTTAATCAATATCATATGGGTATTACTGCTGAAAATTTAGTAGAGCAATATGGTATTTCAAGAGAAGAACAAGATACATTTGCTGTAAACTCACAACAAAAAGCAGTACGTGCACAGCAA |

| **Isolate** | **MLST Allele** | **nt** | **Sequence** |
| --- | --- | --- | --- |
| Gokarna-D ("UNKNOWN 03") | arcC-0001 | 456 | TTATTAATCCAACAAGCTAAATCGAACAGTGACACAACGCCGGCAATGCCATTGGATACTTGTGGTGCAATGTCACAGGGTATGATAGGCTATTGGTTGGAAACTGAAATCAATCGCATTTTAACTGAAATGAATAGTGATAGAACTGTAGGCACAATCGTTACACGTGTGGAAGTAGATAAAGATGATCCACGATTCAATAACCCAACCAAACCAATTGGTCCTTTTTATACGAAAGAAGAAGTTGAAGAATTACAAAAAGAACAGCCAGACTCAGTCTTTAAAGAAGATGCAGGACGTGGTTATAGAAAAGTAGTTGCGTCACCACTACCTCAATCTATACTAGAACACCAGTTAATTCGAACTTTAGCAGACGGTAAAAATATTGTCATTGCATGCGGTGGTGGCGGTATTCCAGTTATAAAAAAAGAAAATACCTATGAAGGTGTTGAAGCG |
|  | aroE-0003 | 456 | AATTTTAATTCTTTAGGATTAGATGATACTTATGAAGCTTTAAATATTCCAATTGAAGATTTTCATTTAATTAAAGAAATTATTTCGAAAAAAGAATTAGAAGGCTTTAATATCACAATTCCTCATAAAGAACGTATCATACCGTATTTAGATTATGTTGATGAACAAGCGATTAATGCAGGTGCAGTTAACACTGTTTTGATAAAAGATGGCAAGTGGATAGGGTATAATACAGATGGTATTGGTTATGTTAAAGGATTGCACAGCGTTTATCCAGATTTAGAAAATGCATACATTTTAATTTTGGGCGCAGGTGGTGCAAGTAAAGGTATTGCTTATGAATTAGCAAAATTTGTAAAGCCCAAATTAACTGTTGCGAATAGAACGATGGCTCGTTTTGAATCTTGGAATTTAAATATAAACCAAATTTCATTAGCAGATGCTGAAAAGTATTTA |
|  | glpF-0001 | 465 | GGTGCTGATTGGATTGTCATCACAGCTGGATGGGGATTAGCGGTTACAATGGGTGTGTTTGCTGTCGGTCAATTCTCAGGTGCACATTTAAACCCAGCGGTGTCTTTAGCTCTTGCATTAGACGGAAGTTTTGATTGGTCATTAGTTCCTGGTTATATTGTTGCTCAAATGTTAGGTGCAATTGTCGGAGCAACAATTGTATGGTTAATGTACTTGCCACATTGGAAAGCGACAGAAGAAGCTGGCGCGAAATTAGGTGTTTTCTCTACAGCACCGGCTATTAAGAATTACTTTGCCAACTTTTTAAGTGAGATTATCGGAACAATGGCATTAACTTTAGGTATTTTATTTATCGGTGTAAACAAAATTGCCGATGGTTTAAATCCTTTAATTGTCGGAGCATTAATTGTTGCAATCGGATTAAGTTTAGGCGGTGCTACTGGTTATGCAATCAACCCAGCACGT |
|  | gmk-0015 | 417 | CGAATATTTGAAGATCCAAGTACATCATATAAGTATTCTATTTCAATGACAACACGTCAAATGCGTGAAGGTGAAGTTGATGGCGTAGATTACTTTTTTAAAACTAGGGATGCGTTTGAAGCTTTAATCAAAGATGACCAATTTATAGAATATGCTGAATATGTAGGCAACTATTATGGTACACCAGTTCAATATGTTAAAGATACAATGGACGAAGGTCATGATGTATTTTTAGAAATTGAAGTAGAAGGTGCAAAGCAAGTTAGAAAGAAATTTCCAGATGCGTTATTTATTTTCTTAGCACCTCCAAGTTTAGATCACTTGAGAGAGCGATTAGTAGGTAGAGGAACAGAATCCAATGAGAAAATACAAAGTCGTATTAACGAAGCGCGTAAAGAAGTTGAAATGATGAATTTA |
|  | pta-0028 | 474 | GCAACACAATTACAAGCAACAGATTATGTTACACCAATCGTGTTAGGTGATGAGACTAAGGTTCAATCTTTAGCGCAAAAACTTGATCTTGATATTTCTAATATTGAATTAATTAATCCTGCGACAAGTGAATTGAAAGCTGAATTAGTCCAATCATTTGTTGAACGACGTAAAGGTAAAGCGACTGAAGAACAAGCGCAAGAATTATTAAACAATGTGAACTACTTCGGTACAATGCTTGTTTATGCTGGTAAAGCAGATGGTTTAGTTAGTGGTGCAGCACATTCAACAGGCGACACTGTGCGTCCAGCTTTACAAATCATCAAAACGAAACCAGGTGTATCAAGAACATCAGGTATCTTCTTTATGATTAAAGGTGATGAACAATACATCTTTGGTGATTGTGCAATCAATCCAGAACTTGATTCACAAGGACTTGCAGAAATTGCAGTAGAAAGTGCAAAATCAGCATTA |
|  | tpi - unknown allele | 402 | CACGAAACAGATGAAGAAATTAACAAAAAAGCGCACGCTATTTTCAAACATGGAATGACTCCAATTATTTGTGTTGGTGAAACAGACGAAGAGCGTGAAAGTGGTAAAGCTAACGATGTTGTAGGTGAGCAAGTTAAGAAAGCTGTTGCAGGTTTATCTGAAGATCAACTTAAATCAGTTGTAATTGCTTATGAGCCAATCTGGGCAATCGGAACTGGTAAATCATCAACATCTGAAGATGCAAATGAAATGTGTGCATTTGTACGTCAAACTATTGCTGACTTATCAAGCAAAGAAGTATCAGAAGCAACTCGTATTCAATATGGTGGTAGTGTTAAACTTAACAACATTAAAGAATACATGGCACAAACTGATATTGATGGGGCATTAGTAGGTGGCGCA |
|  | yqiL-0001 | 516 | GCGTTTAAAGACGTGCCAGCCTATGATTTAGGTGCGACTTTAATAGAACATATTATTAAAGAGACGGGTTTGAATCCAAGTGAGATTGATGAAGTTATCATCGGTAACGTACTACAAGCAGGACAAGGACAAAATCCAGCACGAATTGCTGCTATGAAAGGTGGCTTGCCAGAAACAGTACCTGCATTTACAGTGAATAAAGTATGTGGTTCTGGGTTAAAGTCGATTCAATTAGCATATCAATCTATTGTGACTGGTGAAAATGACATCGTGCTAGCTGGCGGTATGGAGAATATGTCTCAGTCACCAATGCTTGTCAACAACAGTCGCTTCGGTTTTAAAATGGGACATCAATCAATGGTTGATAGCATGGTATATGATGGTTTAACAGATGTATTTAATCAATATCATATGGGTATTACTGCTGAAAATTTAGTGGAGCAATATGGTATTTCAAGAGAAGAACAAGATACATTTGCTGTAAACTCACAACAAAAAGCAGTACGTGCACAGCAA |

| **Isolate** | **MLST Allele** | **nt** | **Sequence** |
| --- | --- | --- | --- |
| Gokarna-F ("UNKNOWN 05") | arcC-0001 | 456 | TTATTAATCCAACAAGCTAAATCGAACAGTGACACAACGCCGGCAATGCCATTGGATACTTGTGGTGCAATGTCACAGGGTATGATAGGCTATTGGTTGGAAACTGAAATCAATCGCATTTTAACTGAAATGAATAGTGATAGAACTGTAGGCACAATCGTTACACGTGTGGAAGTAGATAAAGATGATCCACGATTCAATAACCCAACCAAACCAATTGGTCCTTTTTATACGAAAGAAGAAGTTGAAGAATTACAAAAAGAACAGCCAGACTCAGTCTTTAAAGAAGATGCAGGACGTGGTTATAGAAAAGTAGTTGCGTCACCACTACCTCAATCTATACTAGAACACCAGTTAATTCGAACTTTAGCAGACGGTAAAAATATTGTCATTGCATGCGGTGGTGGCGGTATTCCAGTTATAAAAAAAGAAAATACCTATGAAGGTGTTGAAGCG |
|  | aroE-0001 | 456 | AATTTTAATTCTTTAGGATTAGATGATACTTATGAAGCTTTAAATATTCCAATTGAAGATTTTCATTTAATTAAAGAAATTATTTCGAAAAAAGAATTAGATGGCTTTAATATCACAATTCCTCATAAAGAACGTATCATACCGTATTTAGATCATGTTGATGAACAAGCGATTAATGCAGGTGCAGTTAACACTGTTTTGATAAAAGATGACAAGTGGATAGGGTATAATACAGATGGTATTGGTTATGTTAAAGGATTGCACAGCGTTTATCCAGATTTAGAAAATGCATACATTTTAATTTTGGGCGCAGGTGGTGCAAGTAAAGGTATTGCTTATGAATTAGCAAAATTTGTAAAGCCCAAATTAACTGTTGCGAATAGAACGATGGCTCGTTTTGAATCTTGGAATTTAAATATAAACCAAATTTCATTAGCAGATGCTGAAAAGTATTTA |
|  | glpF-0001 | 465 | GGTGCTGATTGGATTGTCATCACAGCTGGATGGGGATTAGCGGTTACAATGGGTGTGTTTGCTGTCGGTCAATTCTCAGGTGCACATTTAAACCCAGCGGTGTCTTTAGCTCTTGCATTAGACGGAAGTTTTGATTGGTCATTAGTTCCTGGTTATATTGTTGCTCAAATGTTAGGTGCAATTGTCGGAGCAACAATTGTATGGTTAATGTACTTGCCACATTGGAAAGCGACAGAAGAAGCTGGCGCGAAATTAGGTGTTTTCTCTACAGCACCGGCTATTAAGAATTACTTTGCCAACTTTTTAAGTGAGATTATCGGAACAATGGCATTAACTTTAGGTATTTTATTTATCGGTGTAAACAAAATTGCCGATGGTTTAAATCCTTTAATTGTCGGAGCATTAATTGTTGCAATCGGATTAAGTTTAGGCGGTGCTACTGGTTATGCAATCAACCCAGCACGT |
|  | gmk-0001 | 417 | CGAATATTTGAAGATCCAAGTACATCATATAAGTATTCTATTTCAATGACAACACGTCAAATGCGTGAAGGTGAAGTTGATGGCGTAGATTACTTTTTTAAAACTAGGGATGCGTTTGAAGCTTTAATCAAAGATGACCAATTTATAGAATATGCTGAATATGTAGGCAACTATTATGGTACACCAGTTCAATATGTTAAAGATACAATGGACGAAGGTCATGATGTATTTTTAGAAATTGAAGTAGAAGGTGCAAAGCAAGTTAGAAAGAAATTTCCAGATGCGCTATTTATTTTCTTAGCACCTCCAAGTTTAGAACACTTGAGAGAGCGATTAGTAGGTAGAGGAACAGAATCTGATGAGAAAATACAAAGTCGTATTAACGAAGCGCGTAAAGAAGTTGAAATGATGAATTTA |
|  | pta-0028 | 474 | GCAACACAATTACAAGCAACAGATTATGTTACACCAATCGTGTTAGGTGATGAGACTAAGGTTCAATCTTTAGCGCAAAAACTTGATCTTGATATTTCTAATATTGAATTAATTAATCCTGCGACAAGTGAATTGAAAGCTGAATTAGTCCAATCATTTGTTGAACGACGTAAAGGTAAAGCGACTGAAGAACAAGCGCAAGAATTATTAAACAATGTGAACTACTTCGGTACAATGCTTGTTTATGCTGGTAAAGCAGATGGTTTAGTTAGTGGTGCAGCACATTCAACAGGCGACACTGTGCGTCCAGCTTTACAAATCATCAAAACGAAACCAGGTGTATCAAGAACATCAGGTATCTTCTTTATGATTAAAGGTGATGAACAATACATCTTTGGTGATTGTGCAATCAATCCAGAACTTGATTCACAAGGACTTGCAGAAATTGCAGTAGAAAGTGCAAAATCAGCATTA |
|  | tpi-0004 | 402 | CACGAAACAGATGAAGAAATTAACAAAAAAGCGCACGCTATTTTCAAACATGGAATGACTCCAATTATATGTGTTGGTGAAACAGACGAAGAGCGTGAAAGTGGTAAAGCTAACGATGTTGTAGGTGAGCAAGTTAAGAAAGCTGTTGCAGGTTTATCTGAAGATCAACTTAAATCAGTTGTAATTGCTTATGAACCAATCTGGGCAATCGGAACTGGTAAATCATCAACATCTGAAGATGCAAATGAAATGTGTGCATTTGTACGTCAAACTATTGCTGACTTATCAAGCAAAGAAGTATCAGAAGCAACTCGTATTCAATATGGTGGTAGTGTTAAACCTAACAACATTAAAGAATACATGGCACAAACTGATATTGATGGGGCATTAGTAGGTGGCGCA |
|  | yqiL-0011 | 516 | GCGTTTAAAGACGTGCCAGCCTATGATTTAGGTGCGACTTTAATAGAACATATTATTAAAGAGACGGGTTTGAATCCAAGTGAGATTGATGAAGTTATCATCGGTAACGTACTACAAGCAGGACAAGGACAAAATCCAGCACGAATTGCTGCTATGAAAGGTGGCTTGCCAGAAACAGTACCTGCATTTACAGTGAATAAAGTATGTGGTTCTGGGTTAAAGTCGATTCAATTAGCATATCAATCTATTGTGACTGGTGAAAATGACATCGTGCTAGCTGGCGGTATGGAGAATATGTCTCAATCACCAATGCTTGTCAACAACAGTCGCTTTGGTTTTAAAATGGGACATCAATCAATGGTTGATAGCATGGTATATGATGGTTTAACAGATGTATTTAATCAATATCATATGGGTATTACTGCTGAAAATTTAGTAGAGCAATATGGTATTTCAAGAGAAGAACAAGATACATTTGCTGTAAACTCACAACAAAAAGCAGTACGTGCACAGCAA |

| **Isolate** | **MLST Allele** | **nt** | **Sequence** |
| --- | --- | --- | --- |
| Gokarna-54 ("UNKNOWN 06") | arcC-0001 | 456 | TTATTAATCCAACAAGCTAAATCGAACAGTGACACAACGCCGGCAATGCCATTGGATACTTGTGGTGCAATGTCACAGGGTATGATAGGCTATTGGTTGGAAACTGAAATCAATCGCATTTTAACTGAAATGAATAGTGATAGAACTGTAGGCACAATCGTTACACGTGTGGAAGTAGATAAAGATGATCCACGATTCAATAACCCAACCAAACCAATTGGTCCTTTTTATACGAAAGAAGAAGTTGAAGAATTACAAAAAGAACAGCCAGACTCAGTCTTTAAAGAAGATGCAGGACGTGGTTATAGAAAAGTAGTTGCGTCACCACTACCTCAATCTATACTAGAACACCAGTTAATTCGAACTTTAGCAGACGGTAAAAATATTGTCATTGCATGCGGTGGTGGCGGTATTCCAGTTATAAAAAAAGAAAATACCTATGAAGGTGTTGAAGCG |
|  | aroE-0421 | 456 | AATTTTAATTCTTTAGGATTAGATGATACTTATGAAGCTTTAAATATTCCAATTGAAGATTTTCATTTAATTAAAGAAATTATTTCAAAAAAAGAATTAGATGGCTTTAATATCACAATTCCTCATAAAGAACGTATCATATCGTATTTAGATCATGTTGATGAACAAGCGATTAATGCAGGTGCAGTTAACACTGTTTTGATAAAAGATGGCAAGTGGATAGGGTATAATACAGATGGTATTGGTTATGTTAAAGGATTGCACAGCGTTTATCCAGATTTAGAAAATGCATACATTTTAATTTTGGGCGCAGGTGGTGCAAGTAAAGGCATTGCTTATGAATTAGCAAAATTTGTAAAGCCCAAATTAACTGTTGCGAATAGAACGATGGCTCGTTTTGAATCTTGGAATTTAAATATAAACCAAATTTCATTAGCAGATGCTGAAAAGTATTTA |
|  | glpF-0001 | 465 | GGTGCTGATTGGATTGTCATCACAGCTGGATGGGGATTAGCGGTTACAATGGGTGTGTTTGCTGTCGGTCAATTCTCAGGTGCACATTTAAACCCAGCGGTGTCTTTAGCTCTTGCATTAGACGGAAGTTTTGATTGGTCATTAGTTCCTGGTTATATTGTTGCTCAAATGTTAGGTGCAATTGTCGGAGCAACAATTGTATGGTTAATGTACTTGCCACATTGGAAAGCGACAGAAGAAGCTGGCGCGAAATTAGGTGTTTTCTCTACAGCACCGGCTATTAAGAATTACTTTGCCAACTTTTTAAGTGAGATTATCGGAACAATGGCATTAACTTTAGGTATTTTATTTATCGGTGTAAACAAAATTGCCGATGGTTTAAATCCTTTAATTGTCGGAGCATTAATTGTTGCAATCGGATTAAGTTTAGGCGGTGCTACTGGTTATGCAATCAACCCAGCACGT |
|  | gmk - unknown allele | 417 | CGAATATTTGAAGATCCAAGTACATCATATAAGTATTCTATTTCAATGACAACACGTCAAATGCGTGAAGGTGAAGTTGATGGCGTAGATTACTTTTTTAAAACTAGGGATGCGTTTGAAGCTTTAATCAAAGATGACCAATTTATAGAATATGCTGAATATGTAAGCAACTATTATGGTACACCAGTTCAATATGTTAAAGATACAATGGACGAAGGTCATGATGTATTTTTAGAAATTGAAGTAGAAGGTGCAAAGCAAGTTAGAAAGAAATTTCCAGATGCGTTATTTATTTTCTTAGCACCTCCAAGTTTAGATCACTTGAGAGAGCGATTAGTAGGTAGAGGAACAGAATCCGATGAGAAAATACAAAGTCGTATTAACGAAGCACGTAAAGAAGTCGAAATGATGAATTTA |
|  | pta - unknown allele | 474 | GCAACACAATTACAAGCAACAGATTATGTTACACCAATCGTGTTAGGTGATGAGACTAAGGTTCAATCTTTAGCGCAAAAACTTGATCTTGATATTTCTAATATTGAATTAATTAATCCTGCGACAAGTGAATTGAAAGCTGAATTAGTTCAATCATTTGTTGAACGACGCAAAGGTAAAGCGACTGAAGAACAAGCACAAGAATTATTAAACAATGTGAACTACTTCGGTACAATGCTTGTTTATGCTGGTAAAGCAGATGGTTTAGTTAGTGGTGCAGCACATTCAACAGGCGACACTGTGCGTCCAGCTTTACAAATCATCAAAACGAAACCAGGTGTATCAAGAACATCAGGTATCTTCTTTATGATTAAAGGTGATGAACAATACATCTTTGGTGATTGTGCAATCAATCCAGAACTTGATTCACAAGGACTTGCAGAAATTGCAGTAGAAAGTGCAAAATCAGCATTA |
|  | tpi-0001 | 402 | CACGAAACAGATGAAGAAATTAACAAAAAAGCGCACGCTATTTTCAAACATGGAATGACTCCAATTATTTGTGTTGGTGAAACAGACGAAGAGCGTGAAAGTGGTAAAGCTAACGATGTTGTAGGTGAGCAAGTTAAGAAAGCTGTTGCAGGTTTATCTGAAGATCAACTTAAATCAGTTGTAATTGCTTATGAGCCAATCTGGGCAATCGGAACTGGTAAATCATCAACATCTGAAGATGCAAATGAAATGTGTGCATTTGTACGTCAAACTATTGCTGACTTATCAAGCAAAGAAGTATCAGAAGCAACTCGTATTCAATATGGTGGTAGTGTTAAACCTAACAACATTAAAGAATACATGGCACAAACTGATATTGATGGGGCATTAGTAGGTGGCGCA |
|  | yqiL-0011 | 516 | GCGTTTAAAGACGTGCCAGCCTATGATTTAGGTGCGACTTTAATAGAACATATTATTAAAGAGACGGGTTTGAATCCAAGTGAGATTGATGAAGTTATCATCGGTAACGTACTACAAGCAGGACAAGGACAAAATCCAGCACGAATTGCTGCTATGAAAGGTGGCTTGCCAGAAACAGTACCTGCATTTACAGTGAATAAAGTATGTGGTTCTGGGTTAAAGTCGATTCAATTAGCATATCAATCTATTGTGACTGGTGAAAATGACATCGTGCTAGCTGGCGGTATGGAGAATATGTCTCAATCACCAATGCTTGTCAACAACAGTCGCTTTGGTTTTAAAATGGGACATCAATCAATGGTTGATAGCATGGTATATGATGGTTTAACAGATGTATTTAATCAATATCATATGGGTATTACTGCTGAAAATTTAGTAGAGCAATATGGTATTTCAAGAGAAGAACAAGATACATTTGCTGTAAACTCACAACAAAAAGCAGTACGTGCACAGCAA |

| **Isolate** | **MLST Allele** | **nt** | **Sequence** |
| --- | --- | --- | --- |
| Gokarna-51 ("UNKNOWN 07") | arcC-0012 | 456 | TTATTAATCCAACAAGCTAAATCGAACAGTGACACAACGCCGGCAATGCCATTGGATACTTGTGGTGCAATGTCACAGGGTATGATAGGCTATTGGTTGGAAACTGAAATCAATCGCATTTTAACTGAAATGAATAGTGATAGAACCGTAGGCACAATCGTTACACGTGTGGAAGTAGATAAAGATGATCCAAGATTTGATAACCCAACTAAACCAATTGGTCCTTTTTATACGAAAGAAGAAGTTGAAGAATTACAAAAAGAACAGCCAGACTCAGTCTTTAAAGAAGATGCAGGACGTGGTTATAGAAAAGTAGTTGCGTCACCACTACCTCAATCTATACTAGAACACCAGTTAATTCGAACTTTAGCAGACGGTAAAAATATTGTCATTGCATGCGGTGGTGGCGGTATTCCAGTTATAAAAAAAGAAAATACCTATGAAGGTGTTGAAGCG |
|  | aroE - unknown allele | 456 | AATTTTAATTCTTTAGGATTAGATGATACTTATGAAGCTTTAAATATTCCAATTGAAGATTTTCATTTAATTAAAGAAATTATTTCGAAAAAAGAATTAGATGGCTTTAATATCACAATTCCTCATAAAGAACGTATCATACCGTATTTAGATCATGTTGATGAACAAGCGATTAATGCAGGTGCAGTTAACACTGTTTTGATAAAAGATGACAAGTGGATAGGGTATAATACAGATGGTATTGGTTATGTTAAAGGATTGCACAGCGTTTATCCAGATTTAGAAAATGCATACATTTTAATTTTGGGCGCAGGTGGTGCAAGTAAAGGTATTGCTTATGAATTAGCAAAATTTGTAAAGCCCAAATTAACTGTTGCGAATAGAACGATGGCTCGTTTTGAATCTTGGAATTTAAATATAAACCAAATTTCATTGGCAGATGCTGAAAAGTATTTA |
|  | glpF-0001 | 465 | GGTGCTGATTGGATTGTCATCACAGCTGGATGGGGATTAGCGGTTACAATGGGTGTGTTTGCTGTCGGTCAATTCTCAGGTGCACATTTAAACCCAGCGGTGTCTTTAGCTCTTGCATTAGACGGAAGTTTTGATTGGTCATTAGTTCCTGGTTATATTGTTGCTCAAATGTTAGGTGCAATTGTCGGAGCAACAATTGTATGGTTAATGTACTTGCCACATTGGAAAGCGACAGAAGAAGCTGGCGCGAAATTAGGTGTTTTCTCTACAGCACCGGCTATTAAGAATTACTTTGCCAACTTTTTAAGTGAGATTATCGGAACAATGGCATTAACTTTAGGTATTTTATTTATCGGTGTAAACAAAATTGCCGATGGTTTAAATCCTTTAATTGTCGGAGCATTAATTGTTGCAATCGGATTAAGTTTAGGCGGTGCTACTGGTTATGCAATCAACCCAGCACGT |
|  | gmk-0066 | 417 | CGAATATTTGAAGATCCAAGTACATCATATAAGTATTCTATTTCAATGACAACACGTCAAATGCGTGAAGGTGAAGTTGATGGCGTAGATTACTTTTTTAAAACTAGGGATGCGTTTGAAGCTTTAATCAAAGATGACCAATTTATAGAATATGCTGAATATGTAGGCAACTATTATGGTACACCAGTTCAATATGTTAAAGATACAATGGACGAAGGTCATGATGTATTTTTAGAAATTGAAGTAGAAGGTGCAAAGCAAGTTAGAAAGAAATTTCCAGATGCGTTATTTATTTTCTTAGCACCTCCAAGTTTAGATCACTTGAGAGAGCGATTAGTAGGTAGAGGAACAGAATCTGATGAGAAAATACAAAGTCGTATTAACGAAGCGCGTAAAGAAGTTGAAATGATGAATTTA |
|  | pta-0011 | 474 | GCAACACAATTACAAGCAACAGATTATGTTACACCAATCGTGTTAGGTGATGAGACTAAGGTTCAATCTTTAGCGCAAAAACTTAATCTTGATATTTCTAATATTGAATTAATTAATCCTGCGACAAGTGAATTGAAAGCTGAATTAGTCCAATCATTTGTTGAACGACGTAAAGGTAAAGCGACTGAAGAACAAGCGCAAGAATTATTAAACAATGTGAACTACTTCGGTACAATGCTTGTTTATGCTGGTAAAGCAGATGGTTTAGTTAGTGGTGCAGCACATTCAACAGGCGACACTGTGCGTCCAGCTTTACAAATCATCAAAACGAAACCAGGTGTATCAAGAACATCAGGTATCTTCTTTATGATTAAAGGTGATGAACAATACATCTTTGGTGATTGTGCAATCAATCCAGAACTTGATTCACAAGGACTTGCAGAAATTGCAGTAGAAAGTGCAAAATCAGCATTA |
|  | tpi - unknown allele | 402 | CACGAAACAGATGAAGAAATTAACAAAAAAGCGCACGCTATTTTCAAACATGGAATGACTCCAATTATATGTGTTGGTGAAACAGACGAAGAGCGTGAAAGTGGTAAAGCTAACGATGTTGTAGGTGAGCAAGTTAAGAATGCTGTTGCAGGTTTATCTGAAGATCAACTTAAATCAGTTGTAATTGCTTATGAACCAATCTGGGCAATCGGAACTGGTAAATCATCAACATCTGAAGATGCAAATGAAATGTGTGCATTTGTACGTCAAACTATTGCTGACTTATCAAGCAAAGAAGTATCAGAAGCAACTCGTATTCAATATGGTGGTAGTGTTAAACCTAACAACATTAAAGAATACATGGCACAAACTGATATTGATGGGGCATTAGTAGGTGGCGCA |
|  | yqiL - unknown allele | 516 | GCGTTTAAAGACGTGCCAGCCTATGATTTAGGTGCGACTTTAATAGAACATATTATTAAAGAGACGGGTTTGAATCCTAGTGAGATTGATGAAGTTATCATCGGTAACGTACTACAAGCAGGACAAGGACAAAATCCAGCACGAATTGCTGCTATGAAAGGTGGCTTGCCAGAAACAGTACCTGCATTTACGGTGAATAAAGTATGTGGTTCTGGGTTAAAGTCGATTCAATTAGCATATCAATCTATTGTGACTGGTGAAAATGACATCGTGCTAGCTGGCGGTATGGAGAATATGTCTCAGTCACCAATGCTTGTCAACAACAGTCGCTTCGGTTTTAAAATGGGACATCAATCAATGGTTGATAGCATGGTATATGATGGTTTAACAGATGTATTTAATCAATATCATATGGGTATTACTGCTGAAAATTTAGTAGAGCAATATGGTATTTCAAGAGAAGAACAAGATACATTTGCTGTAAACTCACAACAAAAAGCAGTACGTGCACAGCAA |

| **Isolate** | **MLST Allele** | **nt** | **Sequence** |
| --- | --- | --- | --- |
| Ramdi-88 ("UNKNOWN 08") | arcC-0001 | 456 | TTATTAATCCAACAAGCTAAATCGAACAGTGACACAACGCCGGCAATGCCATTGGATACTTGTGGTGCAATGTCACAGGGTATGATAGGCTATTGGTTGGAAACTGAAATCAATCGCATTTTAACTGAAATGAATAGTGATAGAACTGTAGGCACAATCGTTACACGTGTGGAAGTAGATAAAGATGATCCACGATTCAATAACCCAACCAAACCAATTGGTCCTTTTTATACGAAAGAAGAAGTTGAAGAATTACAAAAAGAACAGCCAGACTCAGTCTTTAAAGAAGATGCAGGACGTGGTTATAGAAAAGTAGTTGCGTCACCACTACCTCAATCTATACTAGAACACCAGTTAATTCGAACTTTAGCAGACGGTAAAAATATTGTCATTGCATGCGGTGGTGGCGGTATTCCAGTTATAAAAAAAGAAAATACCTATGAAGGTGTTGAAGCG |
|  | aroE-0038 | 456 | AATTTTAATTCTTTAGGATTAGATGATACTTATGAAGCTTTAAATATTCCAATTGAAGATTTTCATTTAATTAAAGAAATTATTTCGAAAAAAGAATTAGATGGCTTTAATATCACAATTCCTCATAAAGAACGTATCATACCGTATTTAGATCATGTTGATGAACAAGCGATTAATGCAGGTGCAGTTAACACTGTTTTGATAAAAGATGACAAGTGGATAGGGTATAATACAGATGGTATTGGTTATGTTAAAGGATTGCACAGCGTTTATCCAGATTTAGAAAATGCATACATTTTAATTTTGGGCGCAGGTGGTGCAAGTAAAGGCATTGCTTATGAATTAGCAAAATTTGTAAAGCCCAAATTAACTGTTGCGAATAGAACGATGGCTCGTTTTGAATCTTGGAATTTAAATATAAACCAAATTTCATTAGCAGATGCTGAAAAGTATTTA |
|  | glpF-0001 | 465 | GGTGCTGATTGGATTGTCATCACAGCTGGATGGGGATTAGCGGTTACAATGGGTGTGTTTGCTGTCGGTCAATTCTCAGGTGCACATTTAAACCCAGCGGTGTCTTTAGCTCTTGCATTAGACGGAAGTTTTGATTGGTCATTAGTTCCTGGTTATATTGTTGCTCAAATGTTAGGTGCAATTGTCGGAGCAACAATTGTATGGTTAATGTACTTGCCACATTGGAAAGCGACAGAAGAAGCTGGCGCGAAATTAGGTGTTTTCTCTACAGCACCGGCTATTAAGAATTACTTTGCCAACTTTTTAAGTGAGATTATCGGAACAATGGCATTAACTTTAGGTATTTTATTTATCGGTGTAAACAAAATTGCCGATGGTTTAAATCCTTTAATTGTCGGAGCATTAATTGTTGCAATCGGATTAAGTTTAGGCGGTGCTACTGGTTATGCAATCAACCCAGCACGT |
|  | gmk-0001 | 417 | CGAATATTTGAAGATCCAAGTACATCATATAAGTATTCTATTTCAATGACAACACGTCAAATGCGTGAAGGTGAAGTTGATGGCGTAGATTACTTTTTTAAAACTAGGGATGCGTTTGAAGCTTTAATCAAAGATGACCAATTTATAGAATATGCTGAATATGTAGGCAACTATTATGGTACACCAGTTCAATATGTTAAAGATACAATGGACGAAGGTCATGATGTATTTTTAGAAATTGAAGTAGAAGGTGCAAAGCAAGTTAGAAAGAAATTTCCAGATGCGCTATTTATTTTCTTAGCACCTCCAAGTTTAGAACACTTGAGAGAGCGATTAGTAGGTAGAGGAACAGAATCTGATGAGAAAATACAAAGTCGTATTAACGAAGCGCGTAAAGAAGTTGAAATGATGAATTTA |
|  | pta unknown ? or invalid | 476 | GCAACACAATTACAAGCAACAGATTATGTTACACCAATCGTGTTAGGTGATGAGACTAAGGTTCAATCTTTAGCGCAAAAACTTGATCTTGATATTTCTAATATTGAATTAATTAATCCTGCGACAAGTGAATTGAAAGCTGAATTAGTTCAATCATTTGTTGAACGACGTAAAGGTAAAGCGACTGAAGAACAAGCACAAGAATTATTAAACAATGTGAACTACTTCGGTACAATGCTTGTTTATGCTGGTAAAGCAGATGGTTTAGTTAGTGGTGCAAAGCACATTCAACAGGAGACACTGTGCGTCCAGCTTTACAAATCATCAAAACGAAACCAGGTGTATCAAGAACATCAGGTATCTTCTTTATGATTAAAGGTGATGTACAATACATCTTTGGTGATTGTGCAATCAATCCAGAACTTGATTCACAAGGACTTGCAGAAATTGCAGTAGAAAGTGCAAAATCAGCATTA |
|  | tpi-0238 | 402 | CACGAAACAGATGAAGAAATTAACAAAAAAGCGCACGCTATTTTCAAACATGGAATGACGCCAATTATTTGTGTTGGTGAAACAGACGAAGAGCGTGAAAGTGGTAAAGCTAACGATGTTGTAGGTGAGCAAGTTAAGAAAGCTGTTGCAGGTTTATCTGAAGATCAACTTAAATCAGTTGTAATTGCTTATGAACCAATCTGGGCAATCGGAACTGGTAAATCATCAACATCTGAAGATGCAAATGAAATGTGTGCATTTGTACGTCAAACTATTGCTGACTTATCAAGCAAAGAAGTATCAGAAGCAACTCGTATTCAATATGGTGGTAGTGTTAAACCTAACAACATTAAAGAATACATGGCACAAACTGATATTGATGGGGCATTAGTAGGTGGCGCA |
|  | yqiL - unknown allele | 516 | GCGTTTAAAGACGTGCCAGCCTATGATTTAGGTGCGACTTTAATAGAACATATTATTAAAGAGACGGGTTTGAATCCAAGTGAGATTGATGAAGTTATCATCGGTAACGTACTACAAGCAGGACAAGGACAAAATCCAGCACGAATTGCCGCTATGAAAGGTGGCTTGCCAGAAACAGTACCTGCATTTACGGTGAATAAAGTATGTGGTTCTGGGTTAAAGTCGATTCAATTAGCATATCAATCTATTGTGACTGGTGAAAATGACATCGTGCTAGCTGGCGGTATGGAGAATATGTCTCAATCACCAATGCTTGTCAACAACAGTCGCTTTGGTTTTAAAATGGGACATCAATCAATGGTTGATAGCATGGTATATGATGGTTTAACAGATGTATTTAATCAATATCATATGGGTATTACTGCTGAGAATTTAGTAGAACAATATGATATTTCAAGAGAAGAACAAGATACCTTTGCTGTAAACTCACAACAAAAAGCAGTACGTGCACAGCAA |

| **Isolate** | **MLST Allele** | **nt** | **Sequence** |
| --- | --- | --- | --- |
| Pashupati-10 ("UNKNOWN 09") | arcC-0003 | 456 | TTATTAATCCAACAAGCTAAATCGAACAGTGACACAACGCCGGCAATGCCATTGGATACTTGTGGTGCAATGTCACAGGGTATGATAGGCTATTGGTTGGAAACTGAAATCAATCGCATTTTAACTGAAATGAATAGTGATAGAACTGTAGGCACAATCGTTACACGTGTGGAAGTAGATAAAGATGATCCACGATTTGATAACCCAACTAAACCAATTGGTCCTTTTTATACGAAAGAAGAAGTTGAAGAATTACAAAAAGAACAGCCAGACTCAGTCTTTAAAGAAGATGCAGGACGTGGTTATAGAAAAGTAGTTGCGTCACCACTACCTCAATCTATACTAGAACACCAGTTAATTCGAACTTTAGCAGACGGTAAAAATATTGTCATTGCATGCGGTGGTGGCGGTATTCCAGTTATAAAAAAAGAAAATACCTATGAAGGTGTTGAAGCG |
|  | aroE-0003 | 456 | AATTTTAATTCTTTAGGATTAGATGATACTTATGAAGCTTTAAATATTCCAATTGAAGATTTTCATTTAATTAAAGAAATTATTTCGAAAAAAGAATTAGAAGGCTTTAATATCACAATTCCTCATAAAGAACGTATCATACCGTATTTAGATTATGTTGATGAACAAGCGATTAATGCAGGTGCAGTTAACACTGTTTTGATAAAAGATGGCAAGTGGATAGGGTATAATACAGATGGTATTGGTTATGTTAAAGGATTGCACAGCGTTTATCCAGATTTAGAAAATGCATACATTTTAATTTTGGGCGCAGGTGGTGCAAGTAAAGGTATTGCTTATGAATTAGCAAAATTTGTAAAGCCCAAATTAACTGTTGCGAATAGAACGATGGCTCGTTTTGAATCTTGGAATTTAAATATAAACCAAATTTCATTAGCAGATGCTGAAAAGTATTTA |
|  | glpF-0001 | 465 | GGTGCTGATTGGATTGTCATCACAGCTGGATGGGGATTAGCGGTTACAATGGGTGTGTTTGCTGTCGGTCAATTCTCAGGTGCACATTTAAACCCAGCGGTGTCTTTAGCTCTTGCATTAGACGGAAGTTTTGATTGGTCATTAGTTCCTGGTTATATTGTTGCTCAAATGTTAGGTGCAATTGTCGGAGCAACAATTGTATGGTTAATGTACTTGCCACATTGGAAAGCGACAGAAGAAGCTGGCGCGAAATTAGGTGTTTTCTCTACAGCACCGGCTATTAAGAATTACTTTGCCAACTTTTTAAGTGAGATTATCGGAACAATGGCATTAACTTTAGGTATTTTATTTATCGGTGTAAACAAAATTGCCGATGGTTTAAATCCTTTAATTGTCGGAGCATTAATTGTTGCAATCGGATTAAGTTTAGGCGGTGCTACTGGTTATGCAATCAACCCAGCACGT |
|  | gmk-0066 | 417 | CGAATATTTGAAGATCCAAGTACATCATATAAGTATTCTATTTCAATGACAACACGTCAAATGCGTGAAGGTGAAGTTGATGGCGTAGATTACTTTTTTAAAACTAGGGATGCGTTTGAAGCTTTAATCAAAGATGACCAATTTATAGAATATGCTGAATATGTAGGCAACTATTATGGTACACCAGTTCAATATGTTAAAGATACAATGGACGAAGGTCATGATGTATTTTTAGAAATTGAAGTAGAAGGTGCAAAGCAAGTTAGAAAGAAATTTCCAGATGCGTTATTTATTTTCTTAGCACCTCCAAGTTTAGATCACTTGAGAGAGCGATTAGTAGGTAGAGGAACAGAATCTGATGAGAAAATACAAAGTCGTATTAACGAAGCGCGTAAAGAAGTTGAAATGATGAATTTA |
|  | pta-0028 | 474 | GCAACACAATTACAAGCAACAGATTATGTTACACCAATCGTGTTAGGTGATGAGACTAAGGTTCAATCTTTAGCGCAAAAACTTGATCTTGATATTTCTAATATTGAATTAATTAATCCTGCGACAAGTGAATTGAAAGCTGAATTAGTCCAATCATTTGTTGAACGACGTAAAGGTAAAGCGACTGAAGAACAAGCGCAAGAATTATTAAACAATGTGAACTACTTCGGTACAATGCTTGTTTATGCTGGTAAAGCAGATGGTTTAGTTAGTGGTGCAGCACATTCAACAGGCGACACTGTGCGTCCAGCTTTACAAATCATCAAAACGAAACCAGGTGTATCAAGAACATCAGGTATCTTCTTTATGATTAAAGGTGATGAACAATACATCTTTGGTGATTGTGCAATCAATCCAGAACTTGATTCACAAGGACTTGCAGAAATTGCAGTAGAAAGTGCAAAATCAGCATTA |
|  | tpi - unknown allele | 402 | CACGAAACAGATGAAGAAATTAACAAAAAAGCGCACGCTATTTTCAAACATGGAATGACTCCAATTATTTGTGTTGGTGAAACAGACGAAGAGCGTGAAAGTGGTAAAGCTAACGATGTTGTAGGTGAGCAAGTTAAGAAAGCTGTTGCAGGTTTATCTGAAGATCAACTTAAATCAGTTGTAATTGCTTATGAGCCAATCTGGGCAATCGGAACTGGTAAATCATCAACATCTGAAGATGCAAATGAAATGTGTGCATTTGTACGTCAAACTATTGCTGACTTATCAAGCAAAGAAGTATCAGAAGCAACTCGTATTCAATATGGTGGTAGTGTTAAACCTAACAACATTAAAGAATACATGGCACAAACTAATATTGATGGGGCATTAGTAGGTGGCGCA |
|  | yqiL - unknown allele | 516 | GCGTTTAAAGACGTGCCAGCCTATGATTTAGGTGCGACTTTAATAGAACATATTATTAAAGAGACGGGTTTGAATCCTAGTGAGATTGATGAAGTTATCATCGGTAACGTACTACAAGCAGGACAAGGACAAAATCCAGCACGAATTGCTGCTATGAAAGGTGGCTTGCCAGAAACAGTACCTGCATTTACGGTGAATAAAGTATGTGGTTCTGGGTTAAAGTCGATTCAATTAGCATATCAATCTATTGTGACTGGTGAAAATGACATCGTGCTAGCTGGCGGTATGGAGAATATGTCTCAGTCACCAATGCTTGTCAACAACAGTCGCTTCGGTTTTAAAATGGGACATCAATCAATGGTTGATAGCATGGTATATGATGGTTTAACAGATGTATTTAATCAATATCATATGGGTATTACTGCTGAAAATTTAGTAGAGCAATATGGTATTTCAAGAGAAGAACAAGATACATTTGCTGTAAACTCACAACAAAAAGCAGTACGTGCACAGCAA |

| **Isolate** | **MLST Allele** | **nt** | **Sequence** |
| --- | --- | --- | --- |
| Ramdi-90 ("UNKNOWN 10") | arcC-0003 | 456 | TTATTAATCCAACAAGCTAAATCGAACAGTGACACAACGCCGGCAATGCCATTGGATACTTGTGGTGCAATGTCACAGGGTATGATAGGCTATTGGTTGGAAACTGAAATCAATCGCATTTTAACTGAAATGAATAGTGATAGAACTGTAGGCACAATCGTTACACGTGTGGAAGTAGATAAAGATGATCCACGATTTGATAACCCAACTAAACCAATTGGTCCTTTTTATACGAAAGAAGAAGTTGAAGAATTACAAAAAGAACAGCCAGACTCAGTCTTTAAAGAAGATGCAGGACGTGGTTATAGAAAAGTAGTTGCGTCACCACTACCTCAATCTATACTAGAACACCAGTTAATTCGAACTTTAGCAGACGGTAAAAATATTGTCATTGCATGCGGTGGTGGCGGTATTCCAGTTATAAAAAAAGAAAATACCTATGAAGGTGTTGAAGCG |
|  | aroE-0001 | 456 | AATTTTAATTCTTTAGGATTAGATGATACTTATGAAGCTTTAAATATTCCAATTGAAGATTTTCATTTAATTAAAGAAATTATTTCGAAAAAAGAATTAGATGGCTTTAATATCACAATTCCTCATAAAGAACGTATCATACCGTATTTAGATCATGTTGATGAACAAGCGATTAATGCAGGTGCAGTTAACACTGTTTTGATAAAAGATGACAAGTGGATAGGGTATAATACAGATGGTATTGGTTATGTTAAAGGATTGCACAGCGTTTATCCAGATTTAGAAAATGCATACATTTTAATTTTGGGCGCAGGTGGTGCAAGTAAAGGTATTGCTTATGAATTAGCAAAATTTGTAAAGCCCAAATTAACTGTTGCGAATAGAACGATGGCTCGTTTTGAATCTTGGAATTTAAATATAAACCAAATTTCATTAGCAGATGCTGAAAAGTATTTA |
|  | glpF-0001 | 465 | GGTGCTGATTGGATTGTCATCACAGCTGGATGGGGATTAGCGGTTACAATGGGTGTGTTTGCTGTCGGTCAATTCTCAGGTGCACATTTAAACCCAGCGGTGTCTTTAGCTCTTGCATTAGACGGAAGTTTTGATTGGTCATTAGTTCCTGGTTATATTGTTGCTCAAATGTTAGGTGCAATTGTCGGAGCAACAATTGTATGGTTAATGTACTTGCCACATTGGAAAGCGACAGAAGAAGCTGGCGCGAAATTAGGTGTTTTCTCTACAGCACCGGCTATTAAGAATTACTTTGCCAACTTTTTAAGTGAGATTATCGGAACAATGGCATTAACTTTAGGTATTTTATTTATCGGTGTAAACAAAATTGCCGATGGTTTAAATCCTTTAATTGTCGGAGCATTAATTGTTGCAATCGGATTAAGTTTAGGCGGTGCTACTGGTTATGCAATCAACCCAGCACGT |
|  | gmk-0066 | 417 | CGAATATTTGAAGATCCAAGTACATCATATAAGTATTCTATTTCAATGACAACACGTCAAATGCGTGAAGGTGAAGTTGATGGCGTAGATTACTTTTTTAAAACTAGGGATGCGTTTGAAGCTTTAATCAAAGATGACCAATTTATAGAATATGCTGAATATGTAGGCAACTATTATGGTACACCAGTTCAATATGTTAAAGATACAATGGACGAAGGTCATGATGTATTTTTAGAAATTGAAGTAGAAGGTGCAAAGCAAGTTAGAAAGAAATTTCCAGATGCGTTATTTATTTTCTTAGCACCTCCAAGTTTAGATCACTTGAGAGAGCGATTAGTAGGTAGAGGAACAGAATCTGATGAGAAAATACAAAGTCGTATTAACGAAGCGCGTAAAGAAGTTGAAATGATGAATTTA |
|  | pta-0028 | 474 | GCAACACAATTACAAGCAACAGATTATGTTACACCAATCGTGTTAGGTGATGAGACTAAGGTTCAATCTTTAGCGCAAAAACTTGATCTTGATATTTCTAATATTGAATTAATTAATCCTGCGACAAGTGAATTGAAAGCTGAATTAGTCCAATCATTTGTTGAACGACGTAAAGGTAAAGCGACTGAAGAACAAGCGCAAGAATTATTAAACAATGTGAACTACTTCGGTACAATGCTTGTTTATGCTGGTAAAGCAGATGGTTTAGTTAGTGGTGCAGCACATTCAACAGGCGACACTGTGCGTCCAGCTTTACAAATCATCAAAACGAAACCAGGTGTATCAAGAACATCAGGTATCTTCTTTATGATTAAAGGTGATGAACAATACATCTTTGGTGATTGTGCAATCAATCCAGAACTTGATTCACAAGGACTTGCAGAAATTGCAGTAGAAAGTGCAAAATCAGCATTA |
|  | tpi-0001 | 402 | CACGAAACAGATGAAGAAATTAACAAAAAAGCGCACGCTATTTTCAAACATGGAATGACTCCAATTATTTGTGTTGGTGAAACAGACGAAGAGCGTGAAAGTGGTAAAGCTAACGATGTTGTAGGTGAGCAAGTTAAGAAAGCTGTTGCAGGTTTATCTGAAGATCAACTTAAATCAGTTGTAATTGCTTATGAGCCAATCTGGGCAATCGGAACTGGTAAATCATCAACATCTGAAGATGCAAATGAAATGTGTGCATTTGTACGTCAAACTATTGCTGACTTATCAAGCAAAGAAGTATCAGAAGCAACTCGTATTCAATATGGTGGTAGTGTTAAACCTAACAACATTAAAGAATACATGGCACAAACTGATATTGATGGGGCATTAGTAGGTGGCGCA |
|  | yqiL - unknown allele | 516 | GCGTTTAAAGACGTGCCAGCCTATGATTTAGGTGCGACTTTAATAGAACATATTATTAAAGAGACGGGTTTGAATCCTAGTGAGATTGATGAAGTTATCATCGGTAACGTACTACAAGCAGGACAAGGACAAAATCCAGCACGAATTGCTGCTATGAAAGGTGGCTTGCCAGAAACAGTACCTGCATTTACGGTGAATAAAGTATGTGGTTCTGGGTTAAAGTCGATTCAATTAGCATATCAATCTATTGTGACTGGTGAAAATGACATCGTGCTAGCTGGCGGTATGGAGAATATGTCTCAGTCACCAATGCTTGTCAACAACAGTCGCTTCGGTTTTAAAATGGGACATCAATCAATGGTTGATAGCATGGTATATGATGGTTTAACAGATGTATTTAATCAATATCATATGGGTATTACTGCTGAAAATTTAGTAGAGCAATATGGTATTTCAAGAGAAGAACAAGATACATTTGCTGTAAACTCACAACAAAAAGCAGTACGTGCACAGCAA |

| **Isolate** | **MLST Allele** | **nt** | **Sequence** |
| --- | --- | --- | --- |
| Hetauda-61 ("UNKNOWN 12") | arcC-0004 | 456 | TTATTAATCCAACAAGCTAAATCGAACAGTGACACAACGCCGGCAATGCCATTGGATACTTGTGGTGCAATGTCACAGGGTATGATAGGCTATTGGTTGGAAACTGAAATCAATCGCATTTTAACTGAAATGAATAGTGATAGAACCGTAGGCACAATCGTTACACGTGTGGAAGTAGATAAAGATGATCCACGATTCAATAACCCAACCAAACCAATTGGTCCTTTTTATACGAAAGAAGAAGTTGAAGAATTACAAAAAGAACAGCCAGACTCAGTCTTTAAAGAAGATGCAGGACGTGGTTATAGAAAAGTAGTTGCGTCACCACTACCTCAATCTATACTAGAACACCAGTTAATTCGAACTTTAGCAGACGGAAAAAATATTGTCATTGCATGCGGTGGTGGCGGTATTCCAGTTATAAAAAAAGAAAATACCTATGAAGGTGTTGAAGCG |
|  | aroE-0421 | 456 | AATTTTAATTCTTTAGGATTAGATGATACTTATGAAGCTTTAAATATTCCAATTGAAGATTTTCATTTAATTAAAGAAATTATTTCAAAAAAAGAATTAGATGGCTTTAATATCACAATTCCTCATAAAGAACGTATCATATCGTATTTAGATCATGTTGATGAACAAGCGATTAATGCAGGTGCAGTTAACACTGTTTTGATAAAAGATGGCAAGTGGATAGGGTATAATACAGATGGTATTGGTTATGTTAAAGGATTGCACAGCGTTTATCCAGATTTAGAAAATGCATACATTTTAATTTTGGGCGCAGGTGGTGCAAGTAAAGGCATTGCTTATGAATTAGCAAAATTTGTAAAGCCCAAATTAACTGTTGCGAATAGAACGATGGCTCGTTTTGAATCTTGGAATTTAAATATAAACCAAATTTCATTAGCAGATGCTGAAAAGTATTTA |
|  | glpF-0001 | 465 | GGTGCTGATTGGATTGTCATCACAGCTGGATGGGGATTAGCGGTTACAATGGGTGTGTTTGCTGTCGGTCAATTCTCAGGTGCACATTTAAACCCAGCGGTGTCTTTAGCTCTTGCATTAGACGGAAGTTTTGATTGGTCATTAGTTCCTGGTTATATTGTTGCTCAAATGTTAGGTGCAATTGTCGGAGCAACAATTGTATGGTTAATGTACTTGCCACATTGGAAAGCGACAGAAGAAGCTGGCGCGAAATTAGGTGTTTTCTCTACAGCACCGGCTATTAAGAATTACTTTGCCAACTTTTTAAGTGAGATTATCGGAACAATGGCATTAACTTTAGGTATTTTATTTATCGGTGTAAACAAAATTGCCGATGGTTTAAATCCTTTAATTGTCGGAGCATTAATTGTTGCAATCGGATTAAGTTTAGGCGGTGCTACTGGTTATGCAATCAACCCAGCACGT |
|  | gmk-0105 | 417 | CGAATATTTGAAGATCCAAGTACATCATATAAGTATTCTATTTCAATGACAACACGTCAAATGCGTGAAGGTGAAGTTGATGGCGTAGATTACTTTTTTAAAACTAGGGATGCGTTTGAAGCTTTAATCAAAGATGACCAATTTATAGAATATGCTGAATATGTAGGCAACTATTATGGTACACCAGTTCAATATGTTAAAGATACAATGGACGAAGGTCATGATGTATTTTTAGAAATTGAAGTAGAAGGTGCAAAGCAAGTTAGAAAGAAATTTCCAGATGCGTTATTTATTTTCTTAGCACCTCCAAGTTTAGATCACTTGAGAGAGCGATTAGTAGGTAGAGGAACAGAATCCGATGAGAAAATACAAAGTCGTATTAACGAAGCGCGTAAAGAAGTTGAAATGATGAATTTA |
|  | pta-0001 | 474 | GCAACACAATTACAAGCAACAGATTATGTTACACCAATCGTGTTAGGTGATGAGACTAAGGTTCAATCTTTAGCGCAAAAACTTGATCTTGATATTTCTAATATTGAATTAATTAATCCTGCGACAAGTGAATTGAAAGCTGAATTAGTTCAATCATTTGTTGAACGACGTAAAGGTAAAGCGACTGAAGAACAAGCACAAGAATTATTAAACAATGTGAACTACTTCGGTACAATGCTTGTTTATGCTGGTAAAGCAGATGGTTTAGTTAGTGGTGCAGCACATTCAACAGGAGACACTGTGCGTCCAGCTTTACAAATCATCAAAACGAAACCAGGTGTATCAAGAACATCAGGTATCTTCTTTATGATTAAAGGTGATGTACAATACATCTTTGGTGATTGTGCAATCAATCCAGAACTTGATTCACAAGGACTTGCAGAAATTGCAGTAGAAAGTGCAAAATCAGCATTA |
|  | tpi-0005 | 402 | CACGAAACAGATGAAGAAATTAACAAAAAAGCGCACGCTATTTTCAAACATGGAATGACTCCAATTATATGTGTTGGTGAAACAGACGAAGAGCGTGAAAGTGGTAAAGCTAACGATGTTGTAGGTGAGCAAGTTAAGAAAGCTGTTGCAGGTTTATCTGAAAATCAACTAAAATCAGTTGTAATTGCTTATGAACCAATCTGGGCAATCGGAACTGGTAAATCATCAACATCTGAAGATGCGAATGAAATGTGTGCATTTGTACGTCAAACTATTGCTGACTTATCAAGCAAAGAAGTATCAGAAGCAACTCGTATTCAATATGGTGGTAGTGTTAAACCTAACAACATTAAAGAATACATGGCACAAACTGATATTGATGGGGCATTAGTAGGTGGCGCA |
|  | yqiL - unknown allele | 516 | GCGTTTAAAGACGTGCCAGCCTATGATTTAGGTGCGACTTTAATAGAACATATTATTAAAGAGACGGGTTTGAATCCAAGTGAGATTGATGAAGTTATCATCGGTAACGTACTACAAGCAGGACAAGGACAAAATCCAGCACGAATTGCTGCTATGAAAGGTGGCTTGCCAGAAACAGTACCTGCATTTACAGTGAATAAAGTATGTGGTTCTGGGTTAAAGTCGATTCAATTAGCATATCAATCTATTGTGACTGGTGAAAATGACATCGTGCTAGCTGGCGGTATGGAGAATATGTCTCAATCACCAATGCTTGTCAACAACAGTCGCTTCGGTTTTAAAATGGGACATCAATCAATGGTTGATAGCATGGTATATGATGGTTTAACAGATGTATTTAATCAATATCATATGGGTATTACTGCTGAAAATTTAGTAGAGCAATATGGTATTTCAAGAGGAGAACAAGATACATTTGCTGTAAACTCACAACATAAAGCAGTACGTGCACAGCAA |

| **Isolate** | **MLST Allele** | **nt** | **Sequence** |
| --- | --- | --- | --- |
| Hetauda-62 ("UNKNOWN 13") | arcC-0001 | 456 | TTATTAATCCAACAAGCTAAATCGAACAGTGACACAACGCCGGCAATGCCATTGGATACTTGTGGTGCAATGTCACAGGGTATGATAGGCTATTGGTTGGAAACTGAAATCAATCGCATTTTAACTGAAATGAATAGTGATAGAACTGTAGGCACAATCGTTACACGTGTGGAAGTAGATAAAGATGATCCACGATTCAATAACCCAACCAAACCAATTGGTCCTTTTTATACGAAAGAAGAAGTTGAAGAATTACAAAAAGAACAGCCAGACTCAGTCTTTAAAGAAGATGCAGGACGTGGTTATAGAAAAGTAGTTGCGTCACCACTACCTCAATCTATACTAGAACACCAGTTAATTCGAACTTTAGCAGACGGTAAAAATATTGTCATTGCATGCGGTGGTGGCGGTATTCCAGTTATAAAAAAAGAAAATACCTATGAAGGTGTTGAAGCG |
|  | aroE-0003 | 456 | AATTTTAATTCTTTAGGATTAGATGATACTTATGAAGCTTTAAATATTCCAATTGAAGATTTTCATTTAATTAAAGAAATTATTTCGAAAAAAGAATTAGAAGGCTTTAATATCACAATTCCTCATAAAGAACGTATCATACCGTATTTAGATTATGTTGATGAACAAGCGATTAATGCAGGTGCAGTTAACACTGTTTTGATAAAAGATGGCAAGTGGATAGGGTATAATACAGATGGTATTGGTTATGTTAAAGGATTGCACAGCGTTTATCCAGATTTAGAAAATGCATACATTTTAATTTTGGGCGCAGGTGGTGCAAGTAAAGGTATTGCTTATGAATTAGCAAAATTTGTAAAGCCCAAATTAACTGTTGCGAATAGAACGATGGCTCGTTTTGAATCTTGGAATTTAAATATAAACCAAATTTCATTAGCAGATGCTGAAAAGTATTTA |
|  | glpF-0001 | 465 | GGTGCTGATTGGATTGTCATCACAGCTGGATGGGGATTAGCGGTTACAATGGGTGTGTTTGCTGTCGGTCAATTCTCAGGTGCACATTTAAACCCAGCGGTGTCTTTAGCTCTTGCATTAGACGGAAGTTTTGATTGGTCATTAGTTCCTGGTTATATTGTTGCTCAAATGTTAGGTGCAATTGTCGGAGCAACAATTGTATGGTTAATGTACTTGCCACATTGGAAAGCGACAGAAGAAGCTGGCGCGAAATTAGGTGTTTTCTCTACAGCACCGGCTATTAAGAATTACTTTGCCAACTTTTTAAGTGAGATTATCGGAACAATGGCATTAACTTTAGGTATTTTATTTATCGGTGTAAACAAAATTGCCGATGGTTTAAATCCTTTAATTGTCGGAGCATTAATTGTTGCAATCGGATTAAGTTTAGGCGGTGCTACTGGTTATGCAATCAACCCAGCACGT |
|  | gmk - unknown allele | 417 | CGAATATTTGAAGATCCAAGTACATCATATAAGTATTCTATTTCAATGACAACACGTCAAATGCGTGAAGGTGAAGTTGATGGCGTAGATTACTTTTTTAAAACTAGGGATGCGTTTGAAGCTTTAATCAAAGATGACCAATTTATAGAATATGCTGAATATGTAAGCAACTATTATGGTACACCAGTTCAATATGTTAAAGATACAATGGACGAAGGTCATGATGTATTTTTAGAAATTGAAGTAGAAGGTGCAAAGCAAGTTAGAAAGAAATTTCCAGATGCGTTATTTATTTTCTTAGCACCTCCAAGTTTAGATCACTTGAGAGAGCGATTAGTAGGTAGAGGAACAGAATCCGATGAGAAAATACAAAGTCGTATTAACGAAGCACGTAAAGAAGTCGAAATGATGAATTTA |
|  | pta-0001 | 474 | GCAACACAATTACAAGCAACAGATTATGTTACACCAATCGTGTTAGGTGATGAGACTAAGGTTCAATCTTTAGCGCAAAAACTTGATCTTGATATTTCTAATATTGAATTAATTAATCCTGCGACAAGTGAATTGAAAGCTGAATTAGTTCAATCATTTGTTGAACGACGTAAAGGTAAAGCGACTGAAGAACAAGCACAAGAATTATTAAACAATGTGAACTACTTCGGTACAATGCTTGTTTATGCTGGTAAAGCAGATGGTTTAGTTAGTGGTGCAGCACATTCAACAGGAGACACTGTGCGTCCAGCTTTACAAATCATCAAAACGAAACCAGGTGTATCAAGAACATCAGGTATCTTCTTTATGATTAAAGGTGATGTACAATACATCTTTGGTGATTGTGCAATCAATCCAGAACTTGATTCACAAGGACTTGCAGAAATTGCAGTAGAAAGTGCAAAATCAGCATTA |
|  | tpi-0001 | 402 | CACGAAACAGATGAAGAAATTAACAAAAAAGCGCACGCTATTTTCAAACATGGAATGACTCCAATTATTTGTGTTGGTGAAACAGACGAAGAGCGTGAAAGTGGTAAAGCTAACGATGTTGTAGGTGAGCAAGTTAAGAAAGCTGTTGCAGGTTTATCTGAAGATCAACTTAAATCAGTTGTAATTGCTTATGAGCCAATCTGGGCAATCGGAACTGGTAAATCATCAACATCTGAAGATGCAAATGAAATGTGTGCATTTGTACGTCAAACTATTGCTGACTTATCAAGCAAAGAAGTATCAGAAGCAACTCGTATTCAATATGGTGGTAGTGTTAAACCTAACAACATTAAAGAATACATGGCACAAACTGATATTGATGGGGCATTAGTAGGTGGCGCA |
|  | yqiL-0011 | 516 | GCGTTTAAAGACGTGCCAGCCTATGATTTAGGTGCGACTTTAATAGAACATATTATTAAAGAGACGGGTTTGAATCCAAGTGAGATTGATGAAGTTATCATCGGTAACGTACTACAAGCAGGACAAGGACAAAATCCAGCACGAATTGCTGCTATGAAAGGTGGCTTGCCAGAAACAGTACCTGCATTTACAGTGAATAAAGTATGTGGTTCTGGGTTAAAGTCGATTCAATTAGCATATCAATCTATTGTGACTGGTGAAAATGACATCGTGCTAGCTGGCGGTATGGAGAATATGTCTCAATCACCAATGCTTGTCAACAACAGTCGCTTTGGTTTTAAAATGGGACATCAATCAATGGTTGATAGCATGGTATATGATGGTTTAACAGATGTATTTAATCAATATCATATGGGTATTACTGCTGAAAATTTAGTAGAGCAATATGGTATTTCAAGAGAAGAACAAGATACATTTGCTGTAAACTCACAACAAAAAGCAGTACGTGCACAGCAA |

| **Isolate** | **MLST Allele** | **nt** | **Sequence** |
| --- | --- | --- | --- |
| Guheswari-G ("UNKNOWN 14") | arcC-0003 | 456 | TTATTAATCCAACAAGCTAAATCGAACAGTGACACAACGCCGGCAATGCCATTGGATACTTGTGGTGCAATGTCACAGGGTATGATAGGCTATTGGTTGGAAACTGAAATCAATCGCATTTTAACTGAAATGAATAGTGATAGAACTGTAGGCACAATCGTTACACGTGTGGAAGTAGATAAAGATGATCCACGATTTGATAACCCAACTAAACCAATTGGTCCTTTTTATACGAAAGAAGAAGTTGAAGAATTACAAAAAGAACAGCCAGACTCAGTCTTTAAAGAAGATGCAGGACGTGGTTATAGAAAAGTAGTTGCGTCACCACTACCTCAATCTATACTAGAACACCAGTTAATTCGAACTTTAGCAGACGGTAAAAATATTGTCATTGCATGCGGTGGTGGCGGTATTCCAGTTATAAAAAAAGAAAATACCTATGAAGGTGTTGAAGCG |
|  | aroE-0038 | 456 | AATTTTAATTCTTTAGGATTAGATGATACTTATGAAGCTTTAAATATTCCAATTGAAGATTTTCATTTAATTAAAGAAATTATTTCGAAAAAAGAATTAGATGGCTTTAATATCACAATTCCTCATAAAGAACGTATCATACCGTATTTAGATCATGTTGATGAACAAGCGATTAATGCAGGTGCAGTTAACACTGTTTTGATAAAAGATGACAAGTGGATAGGGTATAATACAGATGGTATTGGTTATGTTAAAGGATTGCACAGCGTTTATCCAGATTTAGAAAATGCATACATTTTAATTTTGGGCGCAGGTGGTGCAAGTAAAGGCATTGCTTATGAATTAGCAAAATTTGTAAAGCCCAAATTAACTGTTGCGAATAGAACGATGGCTCGTTTTGAATCTTGGAATTTAAATATAAACCAAATTTCATTAGCAGATGCTGAAAAGTATTTA |
|  | glpF-0001 | 465 | GGTGCTGATTGGATTGTCATCACAGCTGGATGGGGATTAGCGGTTACAATGGGTGTGTTTGCTGTCGGTCAATTCTCAGGTGCACATTTAAACCCAGCGGTGTCTTTAGCTCTTGCATTAGACGGAAGTTTTGATTGGTCATTAGTTCCTGGTTATATTGTTGCTCAAATGTTAGGTGCAATTGTCGGAGCAACAATTGTATGGTTAATGTACTTGCCACATTGGAAAGCGACAGAAGAAGCTGGCGCGAAATTAGGTGTTTTCTCTACAGCACCGGCTATTAAGAATTACTTTGCCAACTTTTTAAGTGAGATTATCGGAACAATGGCATTAACTTTAGGTATTTTATTTATCGGTGTAAACAAAATTGCCGATGGTTTAAATCCTTTAATTGTCGGAGCATTAATTGTTGCAATCGGATTAAGTTTAGGCGGTGCTACTGGTTATGCAATCAACCCAGCACGT |
|  | gmk-0015 | 417 | CGAATATTTGAAGATCCAAGTACATCATATAAGTATTCTATTTCAATGACAACACGTCAAATGCGTGAAGGTGAAGTTGATGGCGTAGATTACTTTTTTAAAACTAGGGATGCGTTTGAAGCTTTAATCAAAGATGACCAATTTATAGAATATGCTGAATATGTAGGCAACTATTATGGTACACCAGTTCAATATGTTAAAGATACAATGGACGAAGGTCATGATGTATTTTTAGAAATTGAAGTAGAAGGTGCAAAGCAAGTTAGAAAGAAATTTCCAGATGCGTTATTTATTTTCTTAGCACCTCCAAGTTTAGATCACTTGAGAGAGCGATTAGTAGGTAGAGGAACAGAATCCAATGAGAAAATACAAAGTCGTATTAACGAAGCGCGTAAAGAAGTTGAAATGATGAATTTA |
|  | pta-0001 | 474 | GCAACACAATTACAAGCAACAGATTATGTTACACCAATCGTGTTAGGTGATGAGACTAAGGTTCAATCTTTAGCGCAAAAACTTGATCTTGATATTTCTAATATTGAATTAATTAATCCTGCGACAAGTGAATTGAAAGCTGAATTAGTTCAATCATTTGTTGAACGACGTAAAGGTAAAGCGACTGAAGAACAAGCACAAGAATTATTAAACAATGTGAACTACTTCGGTACAATGCTTGTTTATGCTGGTAAAGCAGATGGTTTAGTTAGTGGTGCAGCACATTCAACAGGAGACACTGTGCGTCCAGCTTTACAAATCATCAAAACGAAACCAGGTGTATCAAGAACATCAGGTATCTTCTTTATGATTAAAGGTGATGTACAATACATCTTTGGTGATTGTGCAATCAATCCAGAACTTGATTCACAAGGACTTGCAGAAATTGCAGTAGAAAGTGCAAAATCAGCATTA |
|  | tpi - unknown allele | 402 | CACGAAACAGATGAAGAAATTAACAAAAAAGCGCACGCTATTTTCAAACATGGAATGACTCCAATTATATGTGTTGGTGAAACAGACGAAGAGCGTGAAAGTGGTAAAGCTAACGATGTTGTAGGTGAGCAAGTTAAGAAAGCTGTTGCAGGTTTATCTGAAGATCAACTTAAATCAGTTGTAATTGCTTATGAACCAATCTGGGCAATTGGAACTGGTAAATCATCAACATCTGAAGATGCAAATGAAATGTGTGCATTTGTACGTCAAACTATTGCTGACTTATCAAGCAAAGAAGTATCAGAAGCAACTCGTATTCAATATGGTGGTAGTGTTAAACCTAACAACATTAAAGAATACATGGCACAAACTGATATTGATGGGGCATTAGTAGGTGGCGCA |
|  | yqiL-0040 | 516 | GCGTTTAAAGACGTGCCAGCCTATGATTTAGGTGCGACTTTAATAGAACATATTATTAAAGAGACGGGTTTGAATCCAAGTGAGATTGATGAAGTTATCATCGGTAACGTACTACAAGCAGGACAAGGACAAAATCCAGCACGAATTGCTGCTATGAAAGGTGGCTTGCCAGAAACAGTACCTGCATTTACAGTGAATAAAGTATGTGGTTCTGGGTTAAAGTCGATTCAATTAGCATATCAATCTATTGTGACTGGTGAAAATGACATCGTGCTAGCTGGCGGTATGGAGAATATGTCTCAGTCACCAATGCTTGTCAACAACAGTCGCTTCGGTTTTAAAATGGGACATCAATCAATGGTTGATAGCATGGTATATGATGGTTTAACAGATGTATTTAATCAATATCATATGGGTATTACTGCTGAAAATTTAGTAGAGCAATATGGTATTTCAAGAGAAGAACAAGATACATTTGCTGTAAACTCACAACAAAAAGCAGTACGTGCACAGCAA |

| **Isolate** | **MLST Allele** | **nt** | **Sequence** |
| --- | --- | --- | --- |
| Gokarna-E ("UNKNOWN 15") | arcC-0001 | 456 | TTATTAATCCAACAAGCTAAATCGAACAGTGACACAACGCCGGCAATGCCATTGGATACTTGTGGTGCAATGTCACAGGGTATGATAGGCTATTGGTTGGAAACTGAAATCAATCGCATTTTAACTGAAATGAATAGTGATAGAACTGTAGGCACAATCGTTACACGTGTGGAAGTAGATAAAGATGATCCACGATTCAATAACCCAACCAAACCAATTGGTCCTTTTTATACGAAAGAAGAAGTTGAAGAATTACAAAAAGAACAGCCAGACTCAGTCTTTAAAGAAGATGCAGGACGTGGTTATAGAAAAGTAGTTGCGTCACCACTACCTCAATCTATACTAGAACACCAGTTAATTCGAACTTTAGCAGACGGTAAAAATATTGTCATTGCATGCGGTGGTGGCGGTATTCCAGTTATAAAAAAAGAAAATACCTATGAAGGTGTTGAAGCG |
|  | aroE-0421 | 456 | AATTTTAATTCTTTAGGATTAGATGATACTTATGAAGCTTTAAATATTCCAATTGAAGATTTTCATTTAATTAAAGAAATTATTTCAAAAAAAGAATTAGATGGCTTTAATATCACAATTCCTCATAAAGAACGTATCATATCGTATTTAGATCATGTTGATGAACAAGCGATTAATGCAGGTGCAGTTAACACTGTTTTGATAAAAGATGGCAAGTGGATAGGGTATAATACAGATGGTATTGGTTATGTTAAAGGATTGCACAGCGTTTATCCAGATTTAGAAAATGCATACATTTTAATTTTGGGCGCAGGTGGTGCAAGTAAAGGCATTGCTTATGAATTAGCAAAATTTGTAAAGCCCAAATTAACTGTTGCGAATAGAACGATGGCTCGTTTTGAATCTTGGAATTTAAATATAAACCAAATTTCATTAGCAGATGCTGAAAAGTATTTA |
|  | glpF-0001 | 465 | GGTGCTGATTGGATTGTCATCACAGCTGGATGGGGATTAGCGGTTACAATGGGTGTGTTTGCTGTCGGTCAATTCTCAGGTGCACATTTAAACCCAGCGGTGTCTTTAGCTCTTGCATTAGACGGAAGTTTTGATTGGTCATTAGTTCCTGGTTATATTGTTGCTCAAATGTTAGGTGCAATTGTCGGAGCAACAATTGTATGGTTAATGTACTTGCCACATTGGAAAGCGACAGAAGAAGCTGGCGCGAAATTAGGTGTTTTCTCTACAGCACCGGCTATTAAGAATTACTTTGCCAACTTTTTAAGTGAGATTATCGGAACAATGGCATTAACTTTAGGTATTTTATTTATCGGTGTAAACAAAATTGCCGATGGTTTAAATCCTTTAATTGTCGGAGCATTAATTGTTGCAATCGGATTAAGTTTAGGCGGTGCTACTGGTTATGCAATCAACCCAGCACGT |
|  | gmk-0001 | 417 | CGAATATTTGAAGATCCAAGTACATCATATAAGTATTCTATTTCAATGACAACACGTCAAATGCGTGAAGGTGAAGTTGATGGCGTAGATTACTTTTTTAAAACTAGGGATGCGTTTGAAGCTTTAATCAAAGATGACCAATTTATAGAATATGCTGAATATGTAGGCAACTATTATGGTACACCAGTTCAATATGTTAAAGATACAATGGACGAAGGTCATGATGTATTTTTAGAAATTGAAGTAGAAGGTGCAAAGCAAGTTAGAAAGAAATTTCCAGATGCGCTATTTATTTTCTTAGCACCTCCAAGTTTAGAACACTTGAGAGAGCGATTAGTAGGTAGAGGAACAGAATCTGATGAGAAAATACAAAGTCGTATTAACGAAGCGCGTAAAGAAGTTGAAATGATGAATTTA |
|  | pta-0012 | 474 | GCAACACAATTACAAGCAACAGATTATGTTACACCAATCGTGTTAGGTGATGAGACTAAGGTTCAATCTTTAGCGCAAAAACTTGATCTTGATATTTCTAATATTGAATTAATTAATCCTGCGACAAGTGAATTGAAAGCTGAATTAGTTCAATCATTTGTTGAACGACGTAAAGGTAAAGCGACTGAAGAACAAGCACAAGAATTATTAAACAATGTGAACTACTTCGGTACAATGCTTGTTTATGCTGGTAAAGCAGATGGTTTAGTTAGTGGTGCAGCACATTCAACAGGAGACACTGTGCGTCCAGCTTTACAAATCATCAAAACGAAACCAGGTGTATCAAGAACATCAGGTATCTTCTTTATGATTAAAGGTGATGAACAATACATCTTTGGTGATTGTGCAATCAATCCAGAACTTGATTCACAAGGACTTGCAGAAATTGCAGTAGAAAGTGCAAAATCAGCATTA |
|  | tpi-0001 | 402 | CACGAAACAGATGAAGAAATTAACAAAAAAGCGCACGCTATTTTCAAACATGGAATGACTCCAATTATTTGTGTTGGTGAAACAGACGAAGAGCGTGAAAGTGGTAAAGCTAACGATGTTGTAGGTGAGCAAGTTAAGAAAGCTGTTGCAGGTTTATCTGAAGATCAACTTAAATCAGTTGTAATTGCTTATGAGCCAATCTGGGCAATCGGAACTGGTAAATCATCAACATCTGAAGATGCAAATGAAATGTGTGCATTTGTACGTCAAACTATTGCTGACTTATCAAGCAAAGAAGTATCAGAAGCAACTCGTATTCAATATGGTGGTAGTGTTAAACCTAACAACATTAAAGAATACATGGCACAAACTGATATTGATGGGGCATTAGTAGGTGGCGCA |
|  | yqiL-0011 | 516 | GCGTTTAAAGACGTGCCAGCCTATGATTTAGGTGCGACTTTAATAGAACATATTATTAAAGAGACGGGTTTGAATCCAAGTGAGATTGATGAAGTTATCATCGGTAACGTACTACAAGCAGGACAAGGACAAAATCCAGCACGAATTGCTGCTATGAAAGGTGGCTTGCCAGAAACAGTACCTGCATTTACAGTGAATAAAGTATGTGGTTCTGGGTTAAAGTCGATTCAATTAGCATATCAATCTATTGTGACTGGTGAAAATGACATCGTGCTAGCTGGCGGTATGGAGAATATGTCTCAATCACCAATGCTTGTCAACAACAGTCGCTTTGGTTTTAAAATGGGACATCAATCAATGGTTGATAGCATGGTATATGATGGTTTAACAGATGTATTTAATCAATATCATATGGGTATTACTGCTGAAAATTTAGTAGAGCAATATGGTATTTCAAGAGAAGAACAAGATACATTTGCTGTAAACTCACAACAAAAAGCAGTACGTGCACAGCAA |

| **Isolate** | **MLST Allele** | **nt** | **Sequence** |
| --- | --- | --- | --- |
| Guheswari-I ("UNKNOWN 16") | arcC-0003 | 456 | TTATTAATCCAACAAGCTAAATCGAACAGTGACACAACGCCGGCAATGCCATTGGATACTTGTGGTGCAATGTCACAGGGTATGATAGGCTATTGGTTGGAAACTGAAATCAATCGCATTTTAACTGAAATGAATAGTGATAGAACTGTAGGCACAATCGTTACACGTGTGGAAGTAGATAAAGATGATCCACGATTTGATAACCCAACTAAACCAATTGGTCCTTTTTATACGAAAGAAGAAGTTGAAGAATTACAAAAAGAACAGCCAGACTCAGTCTTTAAAGAAGATGCAGGACGTGGTTATAGAAAAGTAGTTGCGTCACCACTACCTCAATCTATACTAGAACACCAGTTAATTCGAACTTTAGCAGACGGTAAAAATATTGTCATTGCATGCGGTGGTGGCGGTATTCCAGTTATAAAAAAAGAAAATACCTATGAAGGTGTTGAAGCG |
|  | aroE-0003 | 456 | AATTTTAATTCTTTAGGATTAGATGATACTTATGAAGCTTTAAATATTCCAATTGAAGATTTTCATTTAATTAAAGAAATTATTTCGAAAAAAGAATTAGAAGGCTTTAATATCACAATTCCTCATAAAGAACGTATCATACCGTATTTAGATTATGTTGATGAACAAGCGATTAATGCAGGTGCAGTTAACACTGTTTTGATAAAAGATGGCAAGTGGATAGGGTATAATACAGATGGTATTGGTTATGTTAAAGGATTGCACAGCGTTTATCCAGATTTAGAAAATGCATACATTTTAATTTTGGGCGCAGGTGGTGCAAGTAAAGGTATTGCTTATGAATTAGCAAAATTTGTAAAGCCCAAATTAACTGTTGCGAATAGAACGATGGCTCGTTTTGAATCTTGGAATTTAAATATAAACCAAATTTCATTAGCAGATGCTGAAAAGTATTTA |
|  | glpF-0001 | 465 | GGTGCTGATTGGATTGTCATCACAGCTGGATGGGGATTAGCGGTTACAATGGGTGTGTTTGCTGTCGGTCAATTCTCAGGTGCACATTTAAACCCAGCGGTGTCTTTAGCTCTTGCATTAGACGGAAGTTTTGATTGGTCATTAGTTCCTGGTTATATTGTTGCTCAAATGTTAGGTGCAATTGTCGGAGCAACAATTGTATGGTTAATGTACTTGCCACATTGGAAAGCGACAGAAGAAGCTGGCGCGAAATTAGGTGTTTTCTCTACAGCACCGGCTATTAAGAATTACTTTGCCAACTTTTTAAGTGAGATTATCGGAACAATGGCATTAACTTTAGGTATTTTATTTATCGGTGTAAACAAAATTGCCGATGGTTTAAATCCTTTAATTGTCGGAGCATTAATTGTTGCAATCGGATTAAGTTTAGGCGGTGCTACTGGTTATGCAATCAACCCAGCACGT |
|  | gmk-0066 | 417 | CGAATATTTGAAGATCCAAGTACATCATATAAGTATTCTATTTCAATGACAACACGTCAAATGCGTGAAGGTGAAGTTGATGGCGTAGATTACTTTTTTAAAACTAGGGATGCGTTTGAAGCTTTAATCAAAGATGACCAATTTATAGAATATGCTGAATATGTAGGCAACTATTATGGTACACCAGTTCAATATGTTAAAGATACAATGGACGAAGGTCATGATGTATTTTTAGAAATTGAAGTAGAAGGTGCAAAGCAAGTTAGAAAGAAATTTCCAGATGCGTTATTTATTTTCTTAGCACCTCCAAGTTTAGATCACTTGAGAGAGCGATTAGTAGGTAGAGGAACAGAATCTGATGAGAAAATACAAAGTCGTATTAACGAAGCGCGTAAAGAAGTTGAAATGATGAATTTA |
|  | pta-0004 | 474 | GCAACACAATTACAAGCAACAGATTATGTTACACCAATCGTGTTAGGTGATGAGACTAAGGTTCAATCTTTAGCGCAAAAACTTGATCTTGATATTTCTAATATTGAATTAATTAATCCTGCGACAAGTGAATTGAAAGCTGAATTAGTTCAATCATTTGTTGAACGACGTAAAGGTAAAGCGACTGAAGAACAAGCACAAGAATTATTAAACAATGTGAACTACTTCGGTACAATGCTTGTTTATGCTGGTAAAGCAGATGGTTTAGTTAGTGGTGCAGCACATTCAACAGGCGACACTGTGCGTCCAGCTTTACAAATCATCAAAACGAAACCAGGTGTATCAAGAACATCAGGTATCTTCTTTATGATTAAAGGTGATGAACAATACATCTTTGGTGATTGTGCAATCAATCCAGAACTTGATTCACAAGGACTTGCAGAAATTGCAGTAGAAAGTGCAAAATCAGCATTA |
|  | tpi-0001 | 402 | CACGAAACAGATGAAGAAATTAACAAAAAAGCGCACGCTATTTTCAAACATGGAATGACTCCAATTATTTGTGTTGGTGAAACAGACGAAGAGCGTGAAAGTGGTAAAGCTAACGATGTTGTAGGTGAGCAAGTTAAGAAAGCTGTTGCAGGTTTATCTGAAGATCAACTTAAATCAGTTGTAATTGCTTATGAGCCAATCTGGGCAATCGGAACTGGTAAATCATCAACATCTGAAGATGCAAATGAAATGTGTGCATTTGTACGTCAAACTATTGCTGACTTATCAAGCAAAGAAGTATCAGAAGCAACTCGTATTCAATATGGTGGTAGTGTTAAACCTAACAACATTAAAGAATACATGGCACAAACTGATATTGATGGGGCATTAGTAGGTGGCGCA |
|  | yqiL - unknown allele | 516 | GCGTTTAAAGACGTGCCAGCCTATGATTTAGGTGCGACTTTATTAGAACATATTATTAAAGAGACGGGTTTGAATCCAAGTGAGATTGATGAAGTTATCATCGGTAACGTACTACAAGCAGGACAAGGACAAAATCCAGCACGAATTGCTGCTATGAAAGGTGGCTTGCCAGAAACAGTACCTGCATTTACAGTGAATAAAGTATGTGGTTCTGGGTTAAAGTCGATTCAATTAGCATATCAATCTATTGTGACTGGTGAAAATGACATCGTGCTAGCTGGCGGTATGGAGAATATGTCTCAATCACCAATGCTTGTCAACAACAGTCGCTTTGGTTTTAAAATGGGACATCAATCAATGGTTGATAGCATGGTATATGATGGTTTAACAGATGTATTTAATCAATATCATATGGGTATTACTGCTGAAAATTTAGTAGAGCAATATGGTATTTCAAGAGAAGAACAAGATACATTTGCTGTAAACTCACAACAAAAAGCAGTACGTGCACAGCAA |
